# Variation in chemotherapy use across healthcare regions in Sweden and its impact on prognosis in women with HR+/HER2- early breast cancer

**DOI:** 10.1186/s13058-026-02366-w

**Published:** 2026-08-13

**Authors:** Balazs Acs, Xingrong Liu, Leo Gkekos, Emelie Karlsson, Anna L. V. Johansson, Per Karlsson, Anne Andersson, Antonios Valachis, Irma Fredriksson, Theodoros Foukakis, Johan Hartman

**Affiliations:** 1 Department of Clinical Pathology and Cancer Diagnostics, Karolinska University Hospital, Stockholm, Sweden; 2 Department of Oncology-Pathology, Karolinska Institutet, Stockholm, Sweden; 3 Department of Medical Epidemiology and Biostatistics, Karolinska Institutet, Stockholm, Sweden; 4 Department of Oncology, Institute of Clinical Sciences, Sahlgrenska Academy and Sahlgrenska University Hospitial, University of Gothenburg, Gothenburg, Sweden; 5 Department of Diagnostics and Interventions, Oncology, Umeå University, Umeå, Sweden; 6 Department of Oncology, Faculty of Medicine and Health, Örebro University, Örebro, Sweden; 7 Breast Center, Karolinska Comprehensive Cancer Center, Stockholm, Sweden; 8 Department of Molecular Medicine and Surgery, Karolinska Institutet, Stockholm, Sweden; 9 Department of Breast-, Endocrine Tumors and Sarcoma, Karolinska Cancer Comprehensive Center, Karolinska University Hospital, Stockholm, Sweden; 10 MedTechLabs, Karolinska University Hospital, BioClinicum, NKS J5:20, Solnavägen, 30, 171 64 Solna, Sweden

**Keywords:** Chemotherapy, Breast cancer, Prognosis, Treatment variation, Real-world data

## Abstract

**Background:**

Hormone receptor–positive/Human epidermal growth factor receptor 2–negative (HR+/HER2–) early breast cancer accounts for the majority of breast cancer cases. Although chemotherapy can reduce recurrence risk, its use in HR+/HER2– high-risk disease remains complex in clinical practice. We aimed to examine regional variation in chemotherapy use in Sweden and its association with prognosis.

**Methods:**

In this population-based cohort study, we included 61,935 women diagnosed with HR+/HER2– early breast cancer between 2007 and 2023, identified through the Swedish National Quality Register for Breast Cancer. Multivariable logistic regression was applied to identify factors that were associated with chemotherapy use across the Swedish healthcare regions. Overall survival was analysed using Kaplan–Meier estimates and regression standardisation based on Cox models, adjusting for clinicopathological and socioeconomic factors.

**Findings:**

Chemotherapy was administered to 28·0% of patients, with substantial regional variation (range 23·0–34·7%). Younger age, larger tumour size, node-positive disease, and higher tumour grade were associated with chemotherapy use. Regional differences in 10-year all-cause mortality persisted after adjustment among patients aged ≥ 65 years but were minimal among those aged < 65 years. The healthcare region with the highest chemotherapy use had the lowest mortality. Socioeconomic factors did not alter these associations.

**Interpretation:**

Chemotherapy use in patients with HR+/HER2– early breast cancer varies across the Swedish healthcare regions despite common guidelines. Among young and middle-aged patients, these differences were not associated with survival disparities. In contrast, among patients aged ≥ 65 years, regional differences in treatment remained associated with differences in prognosis also after socioeconomic adjustments, suggesting undertreatment in some regions. These findings support continued efforts to harmonize implementation of treatment recommendations to ensure equitable care for patients with HR+/HER2 − early breast cancer.

**Supplementary Information:**

The online version contains supplementary material available at 10.1186/s13058-026-02366-w.

## Introduction

Breast cancer remains the most common malignancy among women worldwide, with hormone receptor (HR)-positive and human epidermal growth factor receptor 2 (HER2)-negative subtypes accounting for the majority of early-stage cases [1–3]. Adjuvant chemotherapy (CT) is associated with early risk reduction and improved survival benefits to all patients with HR+/HER2- breast cancer [4–6], although the absolute benefit is dependent on clinicopathological factors and/or gene expression risk profiling results, including assays such as Oncotype DX and Prosigna [7–9].

For patients with intermediate‑risk tumours the magnitude of absolute benefit remains uncertain and falls into a clinical “grey zone” of clinical decision‑making. This therapeutic ambiguity places substantial emphasis on individualised treatment decisions, in which patient preferences, tolerance of treatment toxicity-related and comorbidity burden play important roles to shape treatment choices. Despite national guidelines aiming to standardise care, real-world treatment decisions may vary across healthcare regions due to differences in local clinical practices, resource availability, and physician preferences. Patient preferences further influence decision-making, as the risk–benefit balance of treatment-related harm varies with age and comorbidity burden. Consequently, it remains unclear whether treatment variation in chemotherapy use contributes to regional and age-related disparities in long-term survival.

Sweden’s publicly funded healthcare system, together with the comprehensive and detailed Swedish National Quality Register for Breast Cancer (NKBC), provides a unique opportunity to examine regional variation in cancer care delivery [10, 11]. Previous studies have highlighted disparities in diagnostic pathways and surgical management across Swedish healthcare regions [12, 13], however the extent and impact of regional differences in chemotherapy use for HR+/HER2- early breast cancer remain unclear. Understanding such variation is critical in the context of ongoing efforts to optimise treatment intensity and minimise overtreatment and sometimes persisting side effects in low-risk populations.

In this nationwide, population-based cohort study, we investigated regional variation in chemotherapy use among women diagnosed with HR+/HER2- early breast cancer in Sweden and its impact on long-term survival outcomes. Analyses were further stratified by age and/or treatment setting (neoadjuvant vs adjuvant) to provide clinically relevant insights. We also assessed whether socioeconomic factors, including education and income, confounded these associations. Our findings aim to inform equitable cancer care delivery and guide future policy and clinical decision-making.

## Patients and methods

### Study design and patient population

This is a nationwide cohort study included patients treated for non-metastatic invasive HR+/HER2- breast cancer in Sweden. To evaluate chemotherapy use (administered before or after curative surgery) and regional difference in prognosis, data on patient and tumour characteristics, treatments, hospitals, and follow-up were obtained from the NKBC [11]. Among all female patients diagnosed 2007–2023, we prospectively identified a cohort of women diagnosed with operable non-metastatic, unilateral invasive HR+/HER2- breast cancer. All included patients underwent primary surgery and had reported data on systemic therapy in the register.

Analyses including socioeconomic factors were performed in a subset of the women using the Breast Cancer Data Base Sweden 3.0 (BCBaSe 3.0), an epidemiological research database described previously [14]. In brief, BCBaSe 3.0 includes patients recorded in the NKBC diagnosed between 2008 and 2019, and is individually linked to several national demographic and population-based registers, including the Longitudinal Integrated Database for Health Insurance and Labour Market Studies [15], maintained by Statistics Sweden, enabling the addition of detailed socioeconomic status information.

This study was approved by the Swedish Ethical Review Authority and is reported in accordance with the STROBE guidelines (Strengthening the Reporting of Observational Studies in Epidemiology) statements [16].

### Healthcare regions, covariates and treatments

Based on geographic location, Sweden’s healthcare system is decentralised and organised into six healthcare regions [10]; North, Uppsala-Örebro (Mid-Sweden), Stockholm-Gotland, Southeast, West, and South. National guidelines for breast cancer treatment, however, are standardised across all healthcare regions [17].

Clinical and pathological data were extracted from the NKBC and categorised in groups as follows: age at diagnosis (< 40, 40–49, 50–64, 65–79, and ≥ 80 years), menopausal status (‘premenopausal’ defined in NKBC as < 6 months after last menstruation, ‘postmenopausal’ defined in NKBC ≥ 6 months after last menstruation, or hysterectomy/uncertain), year of diagnosis (2007–2011, 2012–2015, 2016–2019, and 2020–2023), mode of detection (clinically vs screening), tumour size (T1-T4), nodal status (N0, N1-3), TNM classification UICC, 8th edition [18] (I, IIA, IIB, IIIA, and IIIB/IIIC), histological tumour grade (I, II, and III), histological type (ductal, lobular, mixed/others), chemotherapy use (‘Yes’ if administered alone or in combination with other therapies; otherwise as ‘No’), type of curative breast surgery (breast-conserving or mastectomy, including immediate reconstruction), and type of axillary surgery (none, sentinel node biopsy, sampling, or axillary clearance). Information regarding the cause of amenorrhoea, hormonal contraceptive use, hormone-induced bleeding, or bilateral oophorectomy was not available in NKBC. In the subset cohort retrieved from the BCBaSe 3.0, information on level of education (primary education of < 9 years, primary education of 9 years, upper secondary education of < 3 years, upper secondary education of 3 years, tertiary education of < 3 years, tertiary education of ≥ 3 years excluding doctoral studies, and doctoral degree) and the individual sub-component of household disposable income in quintiles (lowest 0–20%, low 20–40%, medium 40–60%, high 60–80%, and highest 80–100%) in the year prior to diagnosis.

Data on systemic therapies, including endocrine therapy and chemotherapy (yes/no), as well as radiotherapy, were extracted along with their timing of relative to surgery. Information on anti-HER2 treatment was obtained to exclude patients with HER2-positive breast cancer. HER2-positivity was defined as HER2 immunohistochemistry staining score 3 + or in-situ hybridisation positivity; if both variables were missing, receipt of anti-HER2 therapy was used as a proxy for HER2-positive status.

### Outcomes

The primary endpoint was overall survival (OS), defined as time from surgery to death from any cause, in accordance with STEEP 2.0 recommendations for studies including patients receiving therapy in neoadjuvant or adjuvant setting [19]. Patients alive at the end of follow-up (until February 13th, 2024) were censored at that date, and those who emigrated were censored at their last recorded date. Chemotherapy use (yes/no) was additionally modelled as a binary outcome to identify factors associated with treatment administration.

### Statistical analysis

Baseline clinicopathological characteristics and treatments were mainly categorised and summarised using frequencies and percentages stratified by multiple scenarios, respectively.

The proportion of patients treated with chemotherapy, was calculated for each healthcare region and visualised on a map of Sweden, with further stratification by age, year of diagnosis, TNM stage, and histological grade. Multivariable logistic regression models were applied to identify patient and tumour characteristics associated with chemotherapy use in routine clinical practice. A fixed-effects approach was applied because the small number of clusters (six healthcare regions) [20], and within-region correlation was assessed using a generalised estimating equation model [21]. Given the evolving changes in indication for neoadjuvant treatment and differences in tumour burden, surgical status, and patient selection between neoadjuvant and adjuvant settings, analyses were also performed separately for each treatment setting. Based on the fitted logistic regression model, pairwise regional comparisons were conducted and depicted, with confidence intervals (or *p*-value) adjusted using the Bonferroni method.

For long-term prognosis across healthcare regions, crude 10-year cumulative risk of all-cause mortality was estimated using the Kaplan–Meier method, and differences between regions were evaluated with the log-rank test. Because the current database did not include breast cancer-specific death, younger patients (< 65 years) with lower comorbidity and fewer competing risks were specifically analysed as a proxy subgroup and compared with patients aged ≥ 65 years to provide clinically relevant insights [22]. In the multivariable analyses, we modelled regional differences in all-cause mortality were conceptualised as arising from two sources: (1) intrinsic regional factors [23, 24] (e.g. healthcare utilisation, clinical expertise, follow-up practices, and socioeconomic contexts), and (2) variation in treatment. Baseline patient and tumour characteristics were included as covariates to account for corresponding differences between patients. To assess differences in prognosis between regions, stepwise adjusted regression models were fitted. **Model 1** included healthcare region and baseline patient and tumour characteristics (age, year of diagnosis, mode of detection, tumour size, nodal status, histological type and grade), to estimate regional differences after case-mix adjustment. **Model 2** additionally incorporated treatment variables to assess whether regional differences in mortality persisted beyond treatment choices, including type of breast and axillary surgery, chemotherapy administration, and timing of chemotherapy (neoadjuvant or adjuvant). **Model 3** further included socioeconomic factors and was restricted to the BCBaSe 3.0 subcohort.

Using regression standardisation approaches based on multivariable models, cumulative risk and incidence rate (per 1000 patient-years) of all-cause mortality across healthcare regions were estimated as marginal association measures [25, 26]. Adjusted cumulative risk of all-cause mortality was estimated using Cox regression models, and Poisson regression was used to estimate crude and adjusted mortality rates. Interaction between healthcare region and chemotherapy use assessed using likelihood ratio tests. A sensitivity analysis restricted to patients diagnosed between 2011 and 2022, when completeness of HER2 status and treatment data was high, was conducted to assess the robustness of the main findings. For Model 3 and relevant comparisons between models and/or cohorts, adjusted hazard ratios (HRs) and 95% confidence intervals (CIs) were reported to illustrate potential variations across different analytical scenarios.

All statistical tests were two-sided, and *p*-value < 0·05 were considered statistically significant. Bonferroni correction was applied for post-hoc pairwise comparisons. Missing data of baseline characteristics were reported as numbers and excluded from percentage calculation. Patients with missing ER-status, HER2-status, and surgical data were excluded from the analysis. Multiple imputation was not performed because the missingness may have been partly attributable to temporal variation in registry coverage, such as early implementation phases and delayed registration, rather than reflecting patient-level characteristics alone. However, sensitivity analyses restricted to the period of 2011–2022, when registry coverage was stable and treatment information was more complete, were conducted to evaluate the robustness of results. Given that missing covariates were present in fewer than 5% of observations, complete case analyses were performed without imputation. Statistical analyses of the NKBC data were conducted using R (version 4.4.2), and the analyses of the BCBaSe 3.0 subgroup data were conducted using STATA (version 18/BE).

### Role of the funding source

The study was conducted independently. The funders did not participate in any aspect of the study, including study design, data collection or extraction, data analysis, interpretation of results, manuscript preparation, and in the decision to submit the manuscript for publication.

## Results

### Study population

**Fig. 1:**
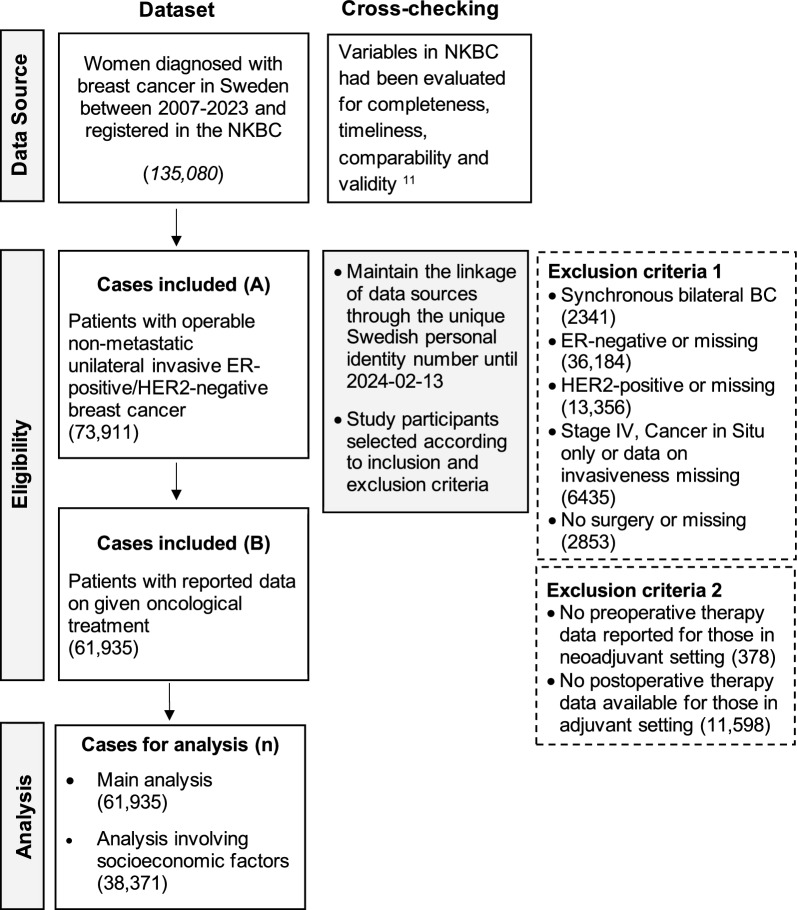
Flow diagram of the study population, prepared in line with the European society for medical oncology guidance for reporting oncology real-world evidence

Of the 135,080 patients registered in the NKBC, 61,935 women with confirmed operable HR+/HER2- early breast cancer were included in the primary cohort (Fig. 1). Baseline patient and tumour characteristics and treatments (Table 1) differed significantly between the healthcare regions (Chi-squared test *p*-value < 0·001). Median age at diagnosis was 65 years (interquartile range 54–72), with a higher proportion of younger patients in the Stockholm-Gotland region (Table 1). In the population-based cohort, 90·2% received endocrine therapy, 56·9% had stage I disease, 71·0% were node-negative (N0), 66·8% had tumours ≤ 2 cm, and 72·8% had invasive carcinoma of no special type (NST; formerly ductal) subtype. Chemotherapy use was more common in younger patients and in those with higher tumour stage, node-positive disease, and higher histological grade (Supplementary Tables 1, 2). Neoadjuvant therapy was increasingly used in recent years among younger patients and those with advanced disease (Supplementary Tables 3–5). A subcohort of 38,371 eligible patients diagnosed between 2008 and 2019 was identified in BCBaSe 3.0 database; 42·0% had upper secondary education (≤ 3 years), and 34·5% of patients in Stockholm-Gotland region were in the highest household disposable income quantile.

**Table 1:** Distribution of baseline characteristics of study participants and tumours in the overall cohort and across six healthcare regions

	Overall cohort n (%)	Healthcare region, n (%)					
		North	Mid Sweden	Stockholm-Gotland	Southeast	West	South
Number of women	61 935	5256 (8.5)	11,347 (18.3)	13,521 (21.8)	7128 (11.5)	13,042 (21.1)	11,641 (18.8)
Age at diagnosis, yr							
Median (IQR)	65 (54–72)	65 (56–72)	65 (55–72)	64 (52–71)	65 (55–72)	64 (54–72)	65 (54–72)
< 40	1588 (2.6)	130 (2.5)	263 (2.3)	423 (3.1)	174 (2.4)	317 (2.4)	281 (2.4)
40–49	8311 (13.4)	578 (11.0)	1331 (11.7)	2105 (15.6)	873 (12.2)	1822 (14.0)	1602 (13.8)
50–64	20,857 (33.7)	1799 (34.2)	3711 (32.7)	4614 (34.1)	2386 (33.5)	4462 (34.2)	3885 (33.4)
65–79	25,503 (41.2)	2299 (43.7)	4959 (43.7)	5374 (39.7)	2903 (40.7)	5212 (40.0)	4756 (40.9)
≥ 80	5676 (9.2)	450 (8.6)	1083 (9.5)	1005 (7.4)	792 (11.1)	1229 (9.4)	1117 (9.6)
Menopausal status							
Premenopausal	11,203 (18.6)	806 (15.7)	1865 (16.9)	3092 (23.2)	1102 (16.0)	2210 (17.7)	2128 (18.6)
Postmenopausal	46,781 (77.5)	4162 (81.2)	8914 (80.5)	10,027 (75.1)	5512 (80.2)	9264 (74.1)	8902 (77.9)
Hysterectomy/uncertain	2374 (3.9)	160 (3.1)	289 (2.6)	233 (1.7)	259 (3.8)	1031 (8.2)	402 (3.5)
Missing	1577	128	279	169	255	537	209
Year of diagnosis							
2007–2011	9048 (14.6)	756 (14.4)	550 (4.8)	1935 (14.3)	1362 (19.1)	2835 (21.7)	1610 (13.8)
2012–2015	18,684 (30.2)	1426 (27.1)	3996 (35.2)	4281 (31.7)	1960 (27.5)	3526 (27.0)	3495 (30.0)
2016–2019	18,825 (30.4)	1687 (32.1)	4000 (35.3)	4189 (31.0)	2031 (28.5)	3494 (26.8)	3424 (29.4)
2020–2023	15,378 (24.8)	1387 (26.4)	2801 (24.7)	3116 (23.0)	1775 (24.9)	3187 (24.4)	3112 (26.7)
Mode of detection							
Clinically detected	27,687 (44.8)	2140 (40.8)	4783 (42.2)	6803 (50.4)	3073 (43.1)	5849 (44.9)	5039 (43.5)
Screening-detected	34,108 (55.2)	3108 (59.2)	6547 (57.8)	6684 (49.6)	4051 (56.9)	7178 (55.1)	6540 (56.5)
Missing	140	8	17	34	4	15	62
Tumour size *							
T0-1	41,289 (66.8)	3611 (68.8)	7734 (68.4)	8994 (66.7)	4476 (62.9)	8319 (63.9)	8155 (70.2)
T2	17,332 (28.1)	1388 (26.5)	2982 (26.4)	3797 (28.1)	2277 (32.0)	3899 (30.0)	2989 (25.7)
T3	2873 (4.6)	200 (3.8)	528 (4.7)	643 (4.8)	336 (4.7)	768 (5.9)	398 (3.4)
T4	293 (0.5)	47 (0.9)	61 (0.5)	58 (0.4)	27 (0.4)	28 (0.2)	72 (0.6)
Missing	148	10	42	29	12	28	27
Lymph node status *							
N0 (negative)	42,900 (71.0)	3647 (71.6)	7855 (71.0)	9716 (72.8)	4937 (71.1)	8930 (71.0)	7815 (68.6)
N + (positive)	17,526 (29.0)	1448 (28.4)	3208 (29.0)	3629 (27.2)	2010 (28.9)	3649 (29.0)	3582 (31.4)
Missing	1509	161	284	176	181	463	244
TNM stage							
I	34,296 (56.9)	2983 (58.7)	6450 (58.5)	7629 (57.3)	3757 (54.2)	6865 (54.7)	6612 (58.1)
IIA	14,295 (23.7)	1145 (22.5)	2484 (22.5)	3245 (24.4)	1741 (25.1)	3048 (24.3)	2632 (23.1)
IIB	6904 (11.5)	539 (10.6)	1163 (10.5)	1551 (11.6)	873 (12.6)	1505 (12.0)	1273 (11.2)
IIIA	3479 (5.8)	298 (5.9)	660 (6.0)	676 (5.1)	403 (5.8)	851 (6.8)	591 (5.2)
IIIB/IIIC	1315 (2.2)	120 (2.4)	270 (2.4)	217 (1.6)	161 (2.3)	284 (2.3)	263 (2.3)
Missing	1646	171	320	203	193	489	270
Histological grade **							
Grade 1	14,738 (24.4)	1267 (24.7)	2961 (26.7)	2819 (21.7)	1719 (24.6)	3242 (25.3)	2730 (24.2)
Grade 2	35,282 (58.5)	2899 (56.6)	6295 (56.7)	7564 (58.2)	4106 (58.7)	7798 (60.8)	6620 (58.7)
Grade 3	10,287 (17.1)	959 (18.7)	1846 (16.6)	2607 (20.1)	1169 (16.7)	1777 (13.9)	1929 (17.1)
Missing	1628	131	245	531	134	225	362
Histological type of BC							
NST (formerly ductal)	45,013 (72.8)	3922 (74.7)	8488 (74.9)	10,100 (74.9)	4880 (68.6)	8984 (69.2)	8639 (74.3)
Lobular	10,143 (16.4)	889 (16.9)	1847 (16.3)	2111 (15.6)	1187 (16.7)	2188 (16.8)	1921 (16.5)
Mixed/other	6655 (10.8)	437 (8.3)	1002 (8.8)	1282 (9.5)	1045 (14.7)	1820 (14.0)	1069 (9.2)
Missing	124	8	10	28	16	50	12
Chemotherapy							
Yes	17,359 (28.0)	1524 (29.0)	2889 (25.5)	4689 (34.7)	2108 (29.6)	3002 (23.0)	3147 (27.0)
No	44,576 (72.0)	3732 (71.0)	8458 (74.5)	8832 (65.3)	5020 (70.4)	10,040 (77.0)	8494 (73.0)
Type of breast surgery							
Breast conserving	42,291 (68.3)	3677 (70.0)	7876 (69.4)	9769 (72.3)	4567 (64.1)	8423 (64.6)	7979 (68.5)
Mastectomy	19,644 (31.7)	1579 (30.0)	3471 (30.6)	3752 (27.7)	2561 (35.9)	4619 (35.4)	3662 (31.5)
Type of axillary surgery							
None	220 (0.4)	13 (0.3)	49 (0.4)	29 (0.2)	19 (0.3)	41 (0.3)	69 (0.6)
SN biopsy	44,204 (72.5)	3737 (72.6)	8379 (75.3)	9858 (73.7)	5069 (72.0)	9004 (70.8)	8157 (70.7)
Sampling	2473 (4.1)	180 (3.5)	382 (3.4)	480 (3.6)	318 (4.5)	632 (5.0)	481 (4.2)
Axillary clearance	14,038 (23.0)	1219 (23.7)	2313 (20.8)	3000 (22.4)	1636 (23.2)	3043 (23.9)	2827 (24.5)
Missing	1000	107	224	154	86	322	107

*Tumor size and lymph node status were categorized by cT/cN at diagnosis or pT/pN after surgery, depending on treatment setting

**Histological grades were evaluated based on the surgical specimen

***Chi-square tests were performed to assess the statistical significance of categorical variable distributions across regions, and all *p*-values were < 0.001

IQR, interquartile range; BC, breast cancer; SN, sentinel node; NST, invasive carcinoma of no special type

### Regional variation in chemotherapy use

**Fig. 2:**
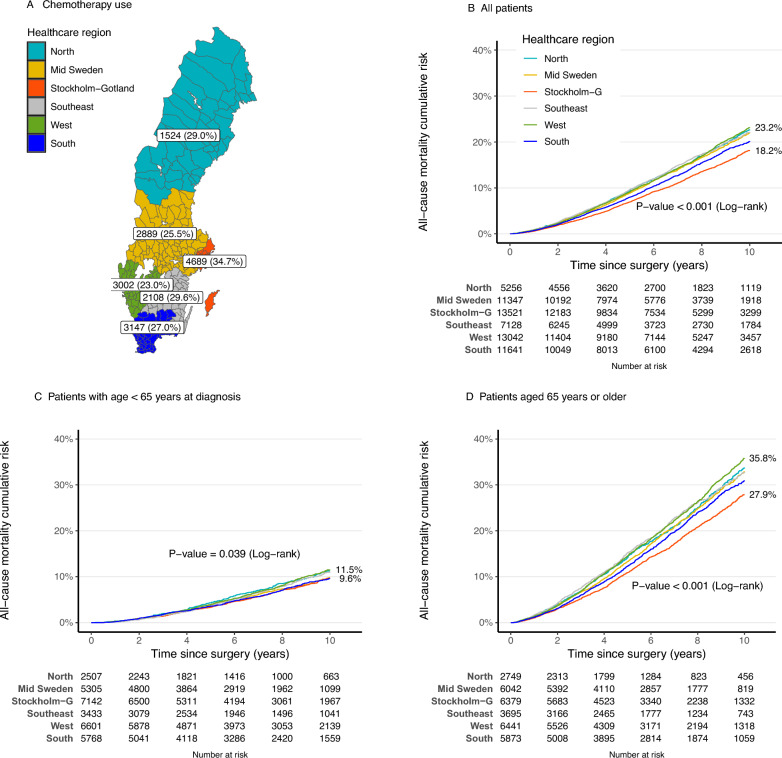
Description of overall chemotherapy use (**A**), with corresponding numbers and percentages across healthcare regions; Unadjusted cumulative risk of all-cause mortality is presented for the overall cohort (**B**), patients younger than 65 years at diagnosis (**C**), and patients aged 65 years or older (**D**), respectively

**Fig. 3:**
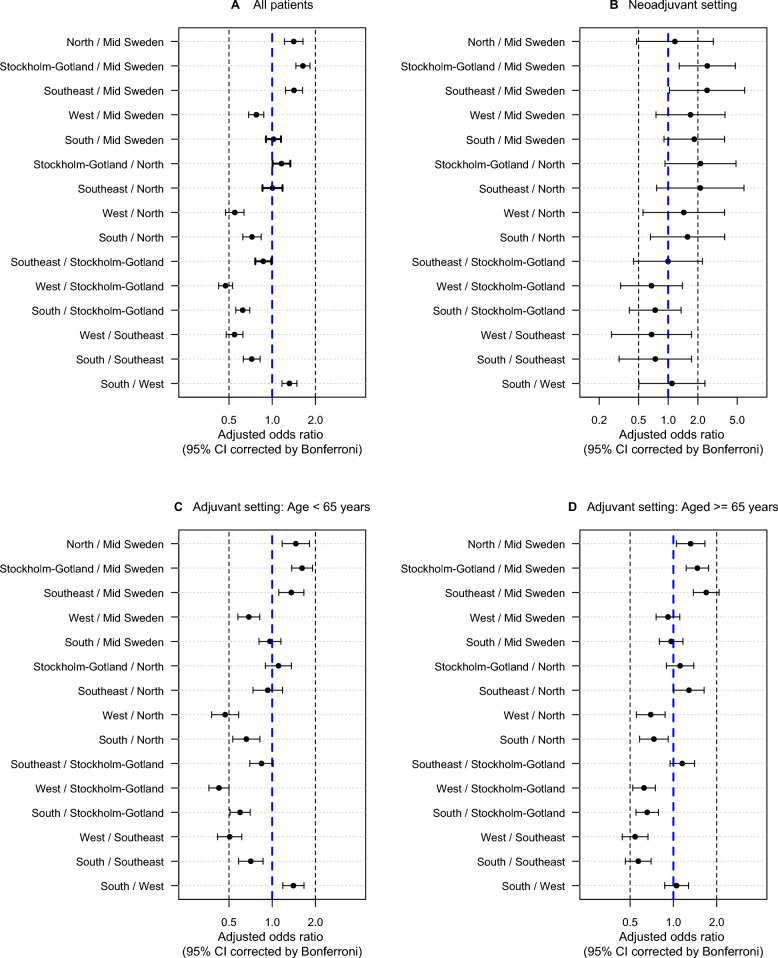
Adjusted odds ratios for pairwise comparison of chemotherapy use across healthcare regions (95% confidence interval, Bonferroni-corrected) are presented for all patients (**A**), patients who received neoadjuvant treatments (**B**), patients younger than 65 years at diagnosis in the adjuvant setting (**C**), and patients aged 65 years or older in the adjuvant settings (**D**)

Overall, 28·0% (17,359) of patients in the study population received chemotherapy, with substantial regional variation between healthcare regions (range 23·0–34·7%; Fig. 2). The Stockholm-Gotland region had the highest chemotherapy use overall, including in the neoadjuvant setting and across both age groups (< 65 years and ≥ 65 years; Fig. 3; Supplementary Table 6; Supplementary Figs. 1, 2). Regional absolute differences were smaller among patients aged 65 years or older than among younger patients (Supplementary Figs. 1, 2). Chemotherapy use decreased with increasing age but increased with higher tumour stage and histological grade, with similar patterns across regions (Supplementary Figs. 3–5). Chemotherapy use remained relatively stable over time from 2011 to 2022, with only minor fluctuations (Supplementary Fig. 6).

**Fig. 4:**
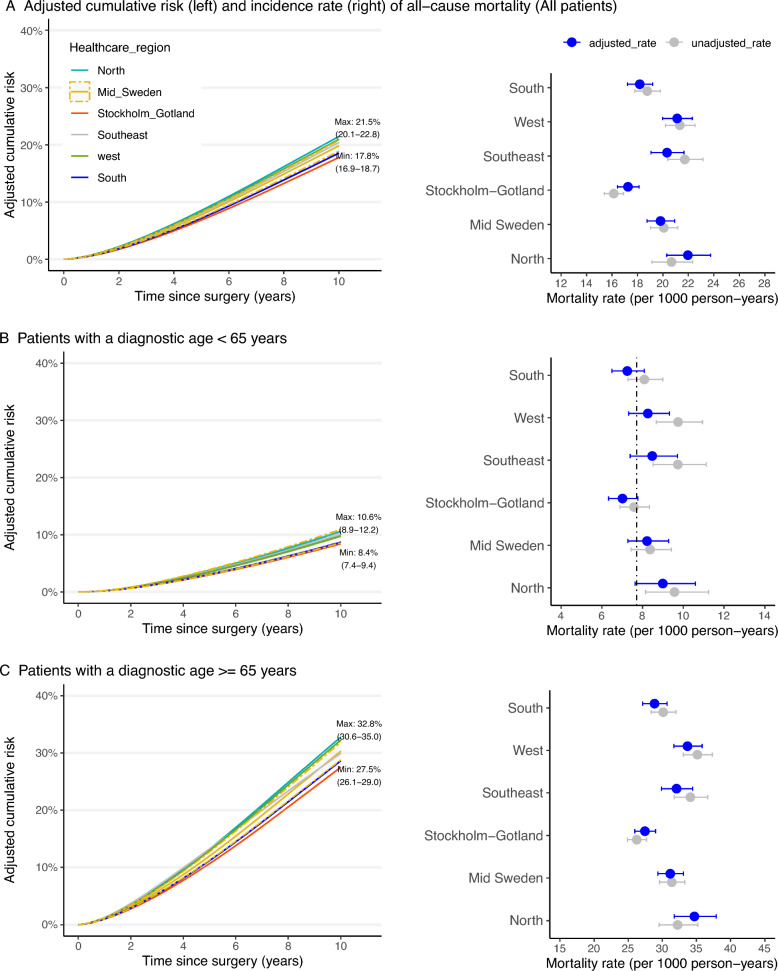
Adjusted (marginal) cumulative risk and incidence rates of all-cause mortality, accounting for patient and tumor characteristics as well as treatments, are presented for the overall cohort (**A**), patients younger than 65 years at diagnosis (**B**), and patients aged 65 years or older (**C**)

Based on the multivariable regression models, the generalised estimating equation approach yielded nearly identical values (by quasi-likelihood information criterion) under both the independent and exchange correlation structures. Subsequently, a standard multivariable logistic regression, incorporating a region indicator variable, showed that neoadjuvant chemotherapy use was significantly associated with tumour characteristics, younger age, more recent year of diagnosis, larger tumour size, and node-positive disease (Table 2). Among patients undergoing primary surgery, adjuvant chemotherapy use was additionally associated with ductal histology and an interaction between tumour grade and mode of detection (likelihood ratio test *p*-value < 0·001; Table 2).

**Table 2:** Multivariable analysis of factors associated with chemotherapy use in neoadjuvant and adjuvant setting, respectively

Factor	Chemotherapy use (0/1)	
	Neoadjuvant setting	Adjuvant setting
	Adjusted OR (95% CI)	Adjusted OR (95% CI)
Age, yrs		
< 40	1 [Reference]	1 [Reference]
40–49	0.137 (0.006–0.744)	0.338 (0.283–0.402)
50–64	0.039 (0.002–0.190)	0.152 (0.128–0.181)
65–79	0.004 (0.0002–0.020)	0.045 (0.038–0.053)
≥ 80	0.0001 (0–0.0005)	0.0004 (0.0003–0.0006)
Year of diagnosis		
2007–2011	1 [Reference]	1 [Reference]
2012–2015	2.12 (1.18–3.75)	1.83 (1.67–2.00)
2016–2019	3.27 (1.85–5.72)	1.86 (1.71–2.03)
2020–2023	2.95 (1.67–5.14)	2.12 (1.94–2.32)
Tumour size ^1^		
T01	1 [Reference]	1 [Reference]
T2	3.42 (2.37–4.94)	2.74 (2.58–2.91)
T3	4.22 (2.69–6.67)	4.69 (4.09–5.37)
T4	5.84 (3.10–11.3)	-
Lymph node status ^1^		
Negative	1 [Reference]	1 [Reference]
Positive	4.62 (3.45–6.25)	10.8 (10.1–11.4)
Interaction term (adjuvant only) ^2^		
Grade 1 and clinically detected	1 [Reference]	1 [Reference]
Grade 2 and clinically detected		4.06 (3.54–4.67)
Grade 3 and clinically detected		24.1 (20.8–28.2)
Grade 1 and screening-detected	1.37 (0.99–1.90)	0.86 (0.73–1.01)
Grade 2 and screening-detected		3.59 (3.13–4.12)
Grade 3 and screening-detected		36.0 (30.9–42.1)
Histological type of breast cancer		
NST (formerly ductal)	1 [Reference]	1 [Reference]
Lobular	0.72 (0.50–1.03)	0.78 (0.73–0.84)
Mixed/other	0.45 (0.27–0.78)	0.81 (0.74–0.90)
Healthcare region^3^		
Mid Sweden	1 [Reference]	1 [Reference]
North	1.17 (0.65–2.14)	1.39 (1.24–1.55)
Stockholm-Gotland	2.49 (1.61–3.85)	1.55 (1.43–1.69)
Southeast	2.48 (1.39–4.50)	1.49 (1.35–1.65)
West	1.68 (0.99–2.89)	0.79 (0.72–0.86)
South	1.84 (1.15–2.95)	0.97 (0.89–1.06)
C-statistic for area under ROC	0.93	0.91

^1^Tumor size and lymph node status were categorized by cT/cN at diagnosis or pT/pN after surgery, depending on treatment setting. In the adjuvant setting, pT34: either pT3 or pT4 (due to less cases in pT4 that may result in unstable estimates of its regression coefficient)

^2^Grade variable was not available in neoadjuvant setting; Likelihood ratio test was used to assess the interaction term with mode of detection in adjuvant setting, with *p*-value < 0.0001

^3^Healthcare region can be treated as one proxy factor of region-level variations

NST, invasive carcinoma of no special type

After adjustment for baseline patient and tumour characteristics, patients diagnosed and treated in the North, Stockholm-Gotland, and Southeast healthcare regions were more likely to receive chemotherapy than those in Mid-Sweden (41%, 64%, and 42%, respectively). Patients in the South had similar likelihood of receiving chemotherapy as those in Mid-Sweden, whereas patients in the West were less likely to receive chemotherapy (Supplementary Table 7); these findings persisted after additional adjustment for socioeconomic factors (Supplementary Table 8a). Regional variation was more pronounced in the adjuvant setting, where regions with higher chemotherapy use were consistently distinguishable from those with lower use across both the entire cohort and the adjuvant subgroup (Fig. 3).

### Regional variation in all-cause mortality

The median follow-up time in the entire cohort was 7·24 years (IQR, 3·99–10·55). The 10-year cumulative risk of all-cause mortality was 22·5% (21·1–24·3), 22·0% (20·9–23·1), 18·2% (17·3–19·1), 22·2% (20·9–23·5), 23·2% (22·2–24·1), and 20·1% (19·1–21·1) in the North, Mid-Sweden, Stockholm-Gotland, Southeast, West, and South healthcare regions, respectively (Fig. 2). Among patients younger than 65 years, the 10-year cumulative risk showed smaller regional variation, ranging from 9·6% (8·6–10·6) in the South to 11·5% (10·5–12·5) in the West. Correspondingly, among those aged 65 years or older, the 10-year cumulative risk ranged from 27·9% (26·4–29·4) in the Stockholm-Gotland region to 35·8% (34·2–37·4) in the West (Fig. 2). Log-rank tests indicated statistically significant differences in long-term all-cause mortality (*p*-value < 0·05).

After adjustment for baseline patient and tumour characteristics (*Model 1*), the standardised (or adjusted) cumulative risk of all-cause mortality at 10-year follow-up in the full cohort ranged from 17·5% (16·7–18·4) in the Stockholm-Gotland region to 21·4% (20·1–22·8) in the North. When both baseline characteristics and treatment factors were included (*Model 2),* the adjusted cumulative risk remained at 17·8% (16·9–18·7) in the Stockholm-Gotland region and 21·5% (20·1–22·8) in the North (Fig. 4). Adding an interaction between region and chemotherapy to *Model 2* did not improve the model fit (test of interaction *p*-value = 0·34) and was therefore not retained in the final model. When restricting the analysis to patients younger than 65 years, regional differences in prognosis diminished after adjustment for baseline patient and tumour characteristics alone and after additional adjustment for treatment factors, suggesting that baseline patient and tumour characteristics were the main drivers of the observed long-term regional differences in prognosis (Fig. 4).

Among patients aged 65 years or older, regional differences in prognosis persisted after adjustment for both baseline characteristics and treatment factors, with the lowest risk of 27·5% (26·1–29·0) in the Stockholm-Gotland region and the highest of 32·8% (30·6–35·0) in the North (Fig. 4). The regional difference in 10-year cumulative risk decreased from 5·9% (region range 27·1–33.0%; Supplementary Fig. 7) in *Model 1* to 5·3% (range 27·5–32·8%) after additional adjustment for treatment factors in *Model 2*, indicating that overall treatment among older patients contributed to the regional differences in prognosis. By comparison, the observed 10-year cumulative risk showed larger regional difference of 7·9% (range 27·9–35·8; Fig. 2), suggesting that baseline characteristics played a substantial role in regional differences in prognosis.

### Regional variation in all-cause mortality after adjustment for socioeconomic factors

Restricting the analysis to women diagnosed between 2008 and 2019 in the BCBaSe 3.0 database, the associations were further adjusted for socioeconomic variables, including education level and disposable income. These adjustments confirmed that the observed regional differences in chemotherapy use and subsequent all-cause mortality were not substantially influenced by socioeconomic status (Supplementary Table 8a and b).

### Sensitivity analysis

The number and percentage of missing ER-status, HER2-status, and non-operated or missing among all patients with unilateral breast cancer were presented in Supplementary Table 9, stratified by healthcare region. The proportion of missing data was similar across regions among patients diagnosed between 2011 and 2022, as well as in the overall cohort including patients diagnosed between 2007 and 2023. The percentage of patients undergoing surgery was slightly lower in 2023, whereas the proportion of patients with confirmed ER-positive, HER2-negative, or operated were stable across year of diagnosis (Supplementary Fig. 8). Unadjusted cumulative risk of mortality remained consistent before and after excluding patients with missing treatment data (Supplementary Fig. 9). In additional sensitivity analyses restricted to patients diagnosed between 2011 and 2022, representing the period with stable registry coverage and complete treatment information, the relevant results were consistent with the main analyses (Supplementary Tables 10, 11; Supplementary Fig. 10).

## Discussion

In this nationwide cohort study of women with HR-positive/HER2-negative early breast cancer, we identified substantial variation in chemotherapy use across Swedish healthcare regions, even after adjustment for case-mix and socioeconomic status. Pairwise regional comparisons showed that similar patterns of variation occurred mainly in the adjuvant setting, irrespective of age. In real-world clinical practice, clinicopathological factors such as younger age, larger tumour size, and node-positive disease were strongly associated with chemotherapy use. Our findings suggest that regional variation in treatment patterns may reflect differences in clinical decision-making, which has previously been reported for comprehensive cancer drugs in Sweden, with documented regional variation [27].

For patients aged < 65 years, who generally have fewer comorbidities and lower non-breast cancer related death [22], long-term prognosis showed limited regional differences in real-world practice. All-cause mortality was statistically similar across regions after adjustment for case-mix*;* however, the pre-specified subgroup analysis was underpowered to detect a statistically significant interaction between region and age < 65 years, and this cutoff may not have been optimal. These findings were also derived from a period in which genomic tests were not routinely used for risk stratification or to identify high-risk disease, which may lead to the potential decline of overtreatment as genomic tests more commonly used today. By contrast, among patients aged 65 years or older, persistent regional differences in all-cause mortality remained, and the region with the highest chemotherapy use had the lowest mortality, even after adjustment for measured case-mix, treatment variation, and socioeconomic status, which was also observed in the overall cohort analysis. Alternatively, regional difference in mortality were assessed separately among premenopausal and postmenopausal women (Supplementary Fig. 11). The statistical significance of the findings was consistent with that observed in the age-startfied analyse (< 65 vs. ≥ 65 years). This suggests that the variability in local clinical decision-making regarding chemotherapy use, including differences in how guidelines are applied to older patients, contributes to the regional differences in prognosis. Other factors, such as comorbidity burden, non-breast cancer related death, or regional health-system characteristics, may also contribute to residual regional differences in prognosis.

Importantly, adjustment for socioeconomic factors such as education and income did not alter the observed associations. This suggests that regional variation in chemotherapy use and long-term outcomes is unlikely to be driven by socioeconomic disparities alone and may instead reflect differences in local clinical traditions, guideline interpretation, or, in more recent years of the study period, in implementation of genomic testing.

Our findings are consistent with previous studies that have examined regional variation in chemotherapy use and its impact on outcomes in early breast cancer. A national data linkage study from Scotland reported modest regional variation in adjuvant chemotherapy rates after adjusting for case-mix, with a trend toward convergence in practice over time, reflecting increasing adherence to risk-stratified treatment guidelines [28]. A Swedish study demonstrated that there is no difference in survival outcomes between HR+/HER2- patients treated with neoadjuvant chemotherapy compared with those treated with adjuvant chemotherapy [29]. However, some Swedish studies have highlighted regional differences in adherence to endocrine therapy and county-level differences in breast cancer survival, suggesting that uniform national guidelines do not always translate into uniform care delivery [12, 30]. Furthermore, a registry-based observational study demonstrated the benefit of chemotherapy use in patients aged 70 years and older with all subtypes of breast cancer and high level of comorbidity [6]. These findings reinforce the importance of evaluating real-world treatment patterns and outcomes to improve care in trial-underrepresented patient groups and to ensure equitable care across regions. Our study has several strengths, including the use of comprehensive national registry data from a country with a structured mammography screening programme for early breast cancer detection and lack of bias in the regional analysis, which enhances the robustness of our results. Regional differences in breast cancer treatment have been observed in many countries. Although the extent of these differences may vary depending on national healthcare system, our findings highlight the importance of ensuring consistent and evidence-based use of chemotherapy following international and national guidelines. However, our study should be interpreted in the context of its observational design, potential residual confounding like healthcare utilisation differences between regions, and lack of data on the temporal evolution of national treatment recommendations and the inability to directly assess guideline concordance using the available registry data. Furthermore, there is a lack of data on patient preferences and comorbidities, which may influence treatment decisions [23]. Another factor that may contribute to the regional variation observed in chemotherapy use is the heterogeneous adoption of genomic profiling assays across Swedish healthcare regions and hospitals. Since 2018, the Swedish national guidelines outline when genomic testing may be considered; however, individual centres differ in whether they routinely use assays such as Prosigna, Oncotype DX, or a region-specific laboratory-developed test assay. Moreover, information on genomic profiling assays has become available in the Swedish National Quality Register for Breast Cancer in 2023. As a result, women with similar tumour characteristics may be assigned different recurrence risk categories depending on the genomic profiling assay used, which may contribute to regional differences in chemotherapy administration between 2018 and 2023. We also found substantial differences in breast cancer grade distribution between regions. Since patients with grade III tumours are considered for adjuvant chemotherapy, this could affect the results. AI-driven computational pathology tools have potential to reduce variability and increase access to independent risk stratification [31, 32]. Another potential contributor to regional differences in chemotherapy use is differential participation in clinical trials and implementation studies evaluating genomic risk stratification and neoadjuvant treatment approaches. Such activities may have facilitated earlier adoption of novel treatment strategies in some centres, albeit these studies are multicentric and not restricted to a single region only. However, information on study participation was not available in the registry and therefore its contribution to the observed regional variation could not be assessed directly.

Comorbidity (e.g., cardiovascular disease, diabetes, etc.) is an important risk factor for older women with breast cancer, and a recent study demonstrated that the relatively low prevalence of multimorbidity in northern Europe is associated with household income [22, 33–35]. Therefore, we validated our main findings in subset of patients for whom socioeconomic factors were available, demonstrating that education and income did not significantly influence the observed differences in chemotherapy use and mortality. By adjusting for socioeconomic factors, we believe that we could also indirectly account, to some extent, for differences in comorbidity. Furthermore, missing data concerning HER2 status and treatment information may have limited the study population, although the proportion of HER2-negative disease remained stable throughout the study period and, robust results were obtained from sensitivity analyses using 2011–2023 data. In addition, the long-term outcome of interest was all-cause mortality rather than breast cancer-specific mortality, which limits the ability to isolate the effect of breast cancer treatment from competing causes of death (e.g., cardiovascular disease, other cancers), particularly among older patients. Moreover, patient reported outcomes should be included to further optimize adjuvant treatment.

Overall, all Swedish residents have universal access to health services; however, geographic inequalities remain and have been addressed in the literature [10, 23]. Similarly, in Norway, Skyrud KD and colleagues found unexplained regional variations in cancer survival persisted, including breast cancer [36], even after adjustment for tumour stage, socioeconomic status, comorbidity, and type of treatment. They argued that the unexplained variations could result from regional inequalities in the quality of cancer care. Future research should explore the role of decision-support tools and multidisciplinary consensus in reducing unwarranted variation and tailoring treatment to identify patients with HR+/HER2- breast cancer who benefit from chemotherapy.

## Conclusions

Differences in chemotherapy use between the Swedish healthcare regions do not impact mortality in patients younger than 65 years with early HR+/HER2- breast cancer. However, among patients aged ≥ 65 years, the persistence of regional differences in prognosis even after socioeconomic adjustments partially reflects local differences in chemotherapy use and undertreatment of patients in some regions. Other contributing factors to the regional differences in prognosis are unmeasured factors such as comorbidity burden, access to supportive social care, non-breast cancer related death, and regional health-system characteristics. Our findings underscore the need for continued efforts to harmonise treatment practices across regions and ensure that chemotherapy is used judiciously, and equitably.

## Supplementary Information

Additional file1

## Data Availability

The data are not publicly available due to restrictions by Swedish and European law, in order to protect patient privacy. Data are available from the register holder of the Swedish National Breast Cancer Quality Register (NKBC) for researchers with relevant ethical approvals and who meet the criteria for access to confidential data.
