# Supplementary material for: Variation in chemotherapy use across healthcare regions in Sweden and its impact on prognosis in women with HR+/HER2- early breast cancer
Source: Breast Cancer Res. 2026 Aug 13;28:139. doi: 10.1186/s13058-026-02366-w (PMC13471642; doi:10.1186/s13058-026-02366-w)
Supplement: Supplementary file 1 [file 13058_2026_2366_MOESM1_ESM.pdf]

Variation in chemotherapy use across healthcare regions in Sweden and its impact on prognosis in women with HR+/HER2- early breast cancer

AUTHORS

Balazs Acs*[1,2], Xingrong Liu*[2], Leo Gkekos[3], Emelie Karlsson[2], Anna L.V. Johansson[3], Per Karlsson[4], Anne Andersson[5], Antonios Valachis[6], Irma Fredriksson[7,8,9], Theodoros Foukakis[2,7#], Johan Hartman[1,2,10#]

Authors' affiliations

[1]Department of Clinical Pathology and Cancer Diagnostics, Karolinska University Hospital, Stockholm, Sweden

[2]Department of Oncology/Pathology, Karolinska Institutet, Stockholm, Sweden

[3]Department of Medical Epidemiology and Biostatistics, Karolinska Institutet, Box 281, SE17177 Stockholm, Sweden.

[4]Department of Oncology, Institute of Clinical Sciences, Sahlgrenska Academy, University of Gothenburg, Gothenburg, Sweden

[5]Department of diagnostics and interventions, oncology, Umeå University, Umeå, Stockholm

[6]Department of Oncology, Faculty of Medicine and Health, Örebro University, Örebro, Sweden

[7]Breast Center, Karolinska Comprehensive Cancer Center, Stockholm, Sweden

[8]Department of Molecular Medicine and Surgery, Karolinska Institutet, Stockholm, Sweden

[9]Department of Breast-, Endocrine Tumors and Sarcoma, Karolinska Cancer Comprehensive Center, Karolinska University Hospital, Stockholm, Sweden

[10]MedTechLabs, Karolinska University Hospital, BioClinicum NKS J5:20, Solnavägen 30, 171 64, Bioclinicum, Solna, Sweden

* Balazs Acs and Xingrong Liu contributed equally as first authors

# Theodoros Foukakis and Johan Hartman contributed equally as last authors

Corresponding author:

Johan Hartman, Professor

Department of Oncology and Pathology, Karolinska Institutet, Stockholm, Sweden;

Department of Clinical Pathology and Cancer Diagnostics, Karolinska University Hospital;

BioClinicum NKS J5:20, Solnavägen 30, 171 64 Solna, Sweden |

Email: johan.hartman@ki.se

Phone: +46707828571

Supplementary Table 1. Distribution of baseline characteristics of the study participants **among those received chemotherapy** and according to 6 healthcare regions, as well as corresponding surgical and systemic therapies

Characteristic	Those received chemotherapy, n (%)	Healthcare region, n (%)					
		North	Mid Sweden	Stockholm-Gotland	Southeast	West	South
**Number of women**	17 359	1524 (8.8)	2889 (16.6)	4689 (27.0)	2108 (12.1)	3002 (17.3)	3147 (18.1)
**Age at diagnosis, yr**							
Median (IQR)	56 (47-65)	58 (48-65)	57 (48-66)	55 (47-64)	58 (49-66)	55 (47-64)	55 (47-65)
< 40	1255 (7.2)	108 (7.1)	210 (7.3)	356 (7.6)	130 (6.2)	223 (7.4)	228 (7.2)
40-49	4318 (24.9)	327 (21.5)	637 (22.0)	1256 (26.8)	448 (21.3)	788 (26.2)	862 (27.4)
50-64	7186 (41.4)	660 (43.3)	1224 (42.4)	1921 (41.0)	876 (41.6)	1255 (41.8)	1250 (39.7)
65-79	4526 (26.1)	423 (27.8)	812 (28.1)	1136 (24.2)	631 (29.9)	726 (24.2)	798 (25.4)
≥ 80	74 (0.4)	6 (0.4)	6 (0.2)	20 (0.4)	23 (1.1)	10 (0.3)	9 (0.3)
**Menopausal status**							
Premenopausal	6041 (36.0)	487 (32.9)	943 (33.8)	1870 (40.4)	589 (29.5)	991 (35.0)	1161 (37.8)
Postmenopausal	9950 (59.2)	930 (62.8)	1756 (62.9)	2649 (57.2)	1297 (65.0)	1558 (55.1)	1760 (57.3)
Hysterectomy/Uncertain	805 (4.8)	64 (4.3)	93 (3.3)	110 (2.4)	110 (5.5)	280 (9.9)	148 (4.8)
Missing	563	43	97	60	112	173	78
**Diagnosis year**							
2007-2011	2249 (13.0)	240 (15.7)	195 (6.7)	671 (14.3)	345 (16.4)	468 (15.6)	330 (10.5)
2012-2015	5214 (30.0)	439 (28.8)	995 (34.4)	1372 (29.3)	569 (27.0)	847 (28.2)	992 (31.5)
2016-2019	5416 (31.2)	505 (33.1)	939 (32.5)	1512 (32.2)	658 (31.2)	811 (27.0)	991 (31.5)
2020-2023	4480 (25.8)	340 (22.3)	760 (26.3)	1134 (24.2)	536 (25.4)	876 (29.2)	834 (26.5)
**Routes to diagnosis**							
Clinically detected	9325 (53.8)	784 (51.5)	1507 (52.2)	2706 (57.9)	1045 (49.6)	1675 (55.9)	1608 (51.3)

Screening-detected	7995 (46.2)	738 (48.5)	1380 (47.8)	1969 (42.1)	1060 (50.4)	1324 (44.1)	1524 (48.7)
Missing	39	2	2	14	3	3	15
**Tumor size** *							
T01	7367 (42.7)	636 (42.0)	1185 (41.3)	2056 (44.0)	846 (40.3)	1074 (36.0)	1570 (50.1)
T2	7910 (45.8)	718 (47.4)	1323 (46.2)	2082 (44.6)	1031 (49.1)	1441 (48.2)	1315 (42.0)
T3	1840 (10.7)	137 (9.0)	326 (11.4)	488 (10.5)	212 (10.1)	460 (15.4)	217 (6.9)
T4	154 (0.9)	25 (1.6)	32 (1.1)	43 (0.9)	12 (0.6)	12 (0.4)	30 (1.0)
Missing	88	8	23	20	7	15	15
**Lymph Node Status** *							
N0 (negative)	6822 (39.6)	581 (38.6)	978 (34.0)	2132 (45.6)	811 (38.8)	1091 (36.7)	1229 (39.6)
N+ (positive)	10400 (60.4)	924 (61.4)	1895 (66.0)	2544 (54.4)	1278 (61.2)	1881 (63.3)	1878 (60.4)
Missing	137	19	16	13	19	30	40
**TNM stage**							
I	3904 (22.8)	288 (19.2)	547 (19.2)	1202 (25.8)	437 (21.0)	561 (19.0)	869 (28.1)
IIA	5675 (33.1)	560 (37.4)	951 (33.3)	1635 (35.1)	714 (34.3)	858 (29.0)	957 (31.0)
IIB	4147 (24.2)	364 (24.3)	697 (24.4)	1110 (23.8)	552 (26.5)	738 (24.9)	686 (22.2)
IIIA	2519 (14.7)	204 (13.6)	487 (17.1)	542 (11.6)	273 (13.1)	604 (20.4)	409 (13.2)
IIIB/IIIC	896 (5.2)	81 (5.4)	171 (6.0)	169 (3.6)	106 (5.1)	198 (6.7)	171 (5.5)
Missing	218	27	36	31	26	43	55
**Histologic Grade (surgery)**							
Grade 1	1113 (6.9)	115 (7.9)	208 (7.6)	326 (7.7)	131 (6.5)	154 (5.4)	179 (6.2)
Grade 2	8846 (54.7)	769 (53.1)	1474 (53.7)	2265 (53.2)	1126 (56.1)	1646 (57.8)	1566 (54.5)
Grade 3	6219 (38.4)	564 (39.0)	1063 (38.7)	1667 (39.1)	751 (37.4)	1047 (36.8)	1127 (39.2)
Missing	1181	76	144	431	100	155	275

**Histologic type of BC**							
NST (formerly ductal)	13353 (77.1)	1168 (76.9)	2186 (75.8)	3679 (78.8)	1553 (73.8)	2295 (76.6)	2472 (78.7)
Lobular	2755 (15.9)	277 (18.2)	495 (17.2)	695 (14.9)	333 (15.8)	464 (15.5)	491 (15.6)
Mixed/other	1209 (7.0)	74 (4.9)	204 (7.1)	297 (6.4)	217 (10.3)	239 (8.0)	178 (5.7)
Missing	42	5	4	18	5	4	6
**Type of breast surgery**							
Breast conserving	9716 (56.0)	875 (57.4)	1562 (54.1)	2878 (61.4)	1112 (52.8)	1527 (50.9)	1762 (56.0)
Mastectomy	7643 (44.0)	649 (42.6)	1327 (45.9)	1811 (38.6)	996 (47.2)	1475 (49.1)	1385 (44.0)
**Type of axillary surgery**							
None	37 (0.2)	2 (0.1)	5 (0.2)	6 (0.1)	4 (0.2)	3 (0.1)	17 (0.5)
SN biopsy	7789 (45.1)	651 (43.1)	1246 (43.3)	2299 (49.1)	913 (43.5)	1264 (42.4)	1416 (45.2)
Sampling	837 (4.8)	45 (3.0)	142 (4.9)	220 (4.7)	123 (5.9)	159 (5.3)	148 (4.7)
Axillary clearance	8624 (49.9)	814 (53.8)	1483 (51.6)	2155 (46.0)	1060 (50.5)	1558 (52.2)	1554 (49.6)
Missing	72	12	13	9	8	18	12

* Tumor size and lymph node status were categorized by cT/cN at diagnosis or pT/pN after surgery, depending on treatment setting.
** Chi-square test for statistical significance of categorical variable distributions across regions were performed with all P-values < 0.001.
Abbreviation: IQR, interquartile range; NST, invasive carcinoma of no special type.

Supplementary Table 2. Distribution of baseline characteristics of the study participants **among those who did not receive any chemotherapy** and according to 6 healthcare regions, as well as corresponding surgical and systemic therapies

**Characteristic**	**Those who did not receive chemotherapy, n (%)**	**Stratified by healthcare regions, n (%)**					
		**North**	**Mid Sweden**	**Stockholm-Gotland**	**Southeast**	**West**	**South**
**Number of women**	44 576	3732 (8.4)	8458 (19.0)	8832 (19.8)	5020 (11.3)	10040 (22.5)	8494 (19.1)
**Age at diagnosis, yr**							
Median (IQR)	67 (59-73)	68 (60-73)	68 (59-74)	67 (58-73)	68 (59-74)	67 (58-73)	67 (59-74)
< 40	333 (0.7)	22 (0.6)	53 (0.6)	67 (0.8)	44 (0.9)	94 (0.9)	53 (0.6)
40-49	3993 (9.0)	251 (6.7)	694 (8.2)	849 (9.6)	425 (8.5)	1034 (10.3)	740 (8.7)
50-64	13671 (30.7)	1139 (30.5)	2487 (29.4)	2693 (30.5)	1510 (30.1)	3207 (31.9)	2635 (31.0)
65-79	20977 (47.1)	1876 (50.3)	4147 (49.0)	4238 (48.0)	2272 (45.3)	4486 (44.7)	3958 (46.6)
≥ 80	5602 (12.6)	444 (11.9)	1077 (12.7)	985 (11.2)	769 (15.3)	1219 (12.1)	1108 (13.0)
**Menopausal status**							
Premenopausal	5162 (11.8)	319 (8.7)	922 (11.1)	1222 (14.0)	513 (10.5)	1219 (12.6)	967 (11.6)
Postmenopausal	36831 (84.5)	3232 (88.6)	7158 (86.5)	7378 (84.6)	4215 (86.4)	7706 (79.6)	7142 (85.4)
Hysterectomy/Uncertain	1569 (3.6)	96 (2.6)	196 (2.4)	123 (1.4)	149 (3.1)	751 (7.8)	254 (3.0)
Missing	1014	85	182	109	143	364	131
**Diagnosis year**							
2007-2011	6799 (15.3)	516 (13.8)	355 (4.2)	1264 (14.3)	1017 (20.3)	2367 (23.6)	1280 (15.1)
2012-2015	13470 (30.2)	987 (26.4)	3001 (35.5)	2909 (32.9)	1391 (27.7)	2679 (26.7)	2503 (29.5)
2016-2019	13409 (30.1)	1182 (31.7)	3061 (36.2)	2677 (30.3)	1373 (27.4)	2683 (26.7)	2433 (28.6)
2020-2023	10898 (24.4)	1047 (28.1)	2041 (24.1)	1982 (22.4)	1239 (24.7)	2311 (23.0)	2278 (26.8)
**Routes to diagnosis**							
Clinically detected	18362 (41.3)	1356 (36.4)	3276 (38.8)	4097 (46.5)	2028 (40.4)	4174 (41.6)	3431 (40.6)
Screening-detected	26113 (58.7)	2370 (63.6)	5167 (61.2)	4715 (53.5)	2991 (59.6)	5854 (58.4)	5016 (59.4)

Missing	101	6	15	20	1	12	47
**Tumor size ***							
T01	33922 (76.2)	2975 (79.8)	6549 (77.6)	6938 (78.6)	3630 (72.4)	7245 (72.3)	6585 (77.6)
T2	9422 (21.2)	670 (18.0)	1659 (19.7)	1715 (19.4)	1246 (24.8)	2458 (24.5)	1674 (19.7)
T3	1033 (2.3)	63 (1.7)	202 (2.4)	155 (1.8)	124 (2.5)	308 (3.1)	181 (2.1)
T4	139 (0.3)	22 (0.6)	29 (0.3)	15 (0.2)	15 (0.3)	16 (0.2)	42 (0.5)
Missing	60	2	19	9	5	13	12
**Lymph Node Status ***							
N0 (negative)	36078 (83.5)	3066 (85.4)	6877 (84.0)	7584 (87.5)	4126 (84.9)	7839 (81.6)	6586 (79.4)
N+ (positive)	7126 (16.5)	524 (14.6)	1313 (16.0)	1085 (12.5)	732 (15.1)	1768 (18.4)	1704 (20.6)
Missing	1372	142	268	163	162	433	204
**TNM stage**							
I	30392 (70.4)	2695 (75.1)	5903 (72.2)	6427 (74.2)	3320 (68.4)	6304 (65.7)	5743 (69.4)
IIA	8620 (20.0)	585 (16.3)	1533 (18.8)	1610 (18.6)	1027 (21.2)	2190 (22.8)	1675 (20.2)
IIB	2757 (6.4)	175 (4.9)	466 (5.7)	441 (5.1)	321 (6.6)	767 (8.0)	587 (7.1)
IIIA	960 (2.2)	94 (2.6)	173 (2.1)	134 (1.5)	130 (2.7)	247 (2.6)	182 (2.2)
IIIB/IIIC	419 (1.0)	39 (1.1)	99 (1.2)	48 (0.6)	55 (1.1)	86 (0.9)	92 (1.1)
Missing	1428	144	284	172	167	446	215
**Histologic Grade (surgery)**							
Grade 1	13625 (30.9)	1152 (31.3)	2753 (32.9)	2493 (28.6)	1588 (31.8)	3088 (31.0)	2551 (30.3)
Grade 2	26436 (59.9)	2130 (57.9)	4821 (57.7)	5299 (60.7)	2980 (59.8)	6152 (61.7)	5054 (60.1)
Grade 3	4068 (9.2)	395 (10.7)	783 (9.4)	940 (10.8)	418 (8.4)	730 (7.3)	802 (9.5)
Missing	447	55	101	100	34	70	87
**Histologic type of BC**							

NST (formerly ductal)	31660 (71.2)	2754 (73.9)	6302 (74.6)	6421 (72.8)	3327 (66.4)	6689 (66.9)	6167 (72.7)
Lobular	7388 (16.6)	612 (16.4)	1352 (16.0)	1416 (16.1)	854 (17.0)	1724 (17.3)	1430 (16.8)
Mixed/other	5446 (12.2)	363 (9.7)	798 (9.4)	985 (11.2)	828 (16.5)	1581 (15.8)	891 (10.5)
Missing	82	3	6	10	11	46	6
**Type of breast surgery**							
Breast conserving	32575 (73.1)	2802 (75.1)	6314 (74.7)	6891 (78.0)	3455 (68.8)	6896 (68.7)	6217 (73.2)
Mastectomy	12001 (26.9)	930 (24.9)	2144 (25.3)	1941 (22.0)	1565 (31.2)	3144 (31.3)	2277 (26.8)
**Type of axillary surgery**							
None	183 (0.4)	11 (0.3)	44 (0.5)	23 (0.3)	15 (0.3)	38 (0.4)	52 (0.6)
SN biopsy	36415 (83.4)	3086 (84.9)	7133 (86.5)	7559 (87.0)	4156 (84.1)	7740 (79.5)	6741 (80.3)
Sampling	1636 (3.7)	135 (3.7)	240 (2.9)	260 (3.0)	195 (3.9)	473 (4.9)	333 (4.0)
Axillary clearance	5414 (12.4)	405 (11.1)	830 (10.1)	845 (9.7)	576 (11.7)	1485 (15.3)	1273 (15.2)
Missing	928	95	211	145	78	304	95

* Tumor size and lymph node status were categorized by cT/cN at diagnosis or pT/pN after surgery, depending on treatment setting.
** Chi-square test for statistical significance of categorical variable distributions across regions were performed with all P-values < 0.001.
Abbreviation: IQR, interquartile range; NST, invasive carcinoma of no special type

**Supplementary Table 3.** Distribution of baseline characteristics of the study participants in those aged < 65 years and according to 6 healthcare regions, as well as corresponding medical and surgical therapies							
**Characteristics**	**Those aged < 65 years, n (%)**	**Healthcare region, n (%)**					
		**North**	**Mid Sweden**	**Stockholm-Gotland**	**Southeast**	**West**	**South**
**Number of women**	30 756	2507 (8.2)	5305 (17.2)	7142 (23.2)	3433 (11.2)	6601 (21.5)	5768 (18.8)
**Age at diagnosis, yr**							
Median (IQR), yr	54 (48-60)	56 (49-61)	55 (48-60)	53 (47-60)	54 (48-60)	54 (48-60)	54 (48-60)
< 40	1588 (5.2)	130 (5.2)	263 (5.0)	423 (5.9)	174 (5.1)	317 (4.8)	281 (4.9)
40-49	8311 (27.0)	578 (23.1)	1331 (25.1)	2105 (29.5)	873 (25.4)	1822 (27.6)	1602 (27.8)
50-64	20857 (67.8)	1799 (71.8)	3711 (70.0)	4614 (64.6)	2386 (69.5)	4462 (67.6)	3885 (67.4)
**Menopausal status**							
Premenopausal	11146 (37.9)	797 (33.1)	1851 (36.7)	3085 (44.1)	1097 (34.2)	2204 (35.8)	2112 (37.8)
Postmenopausal	16218 (55.2)	1471 (61.1)	2916 (57.9)	3712 (53.0)	1865 (58.2)	3141 (51.1)	3113 (55.7)
Hysterectomy/Uncertain	2023 (6.9)	140 (5.8)	270 (5.4)	204 (2.9)	242 (7.6)	804 (13.1)	363 (6.5)
Missing	1369	99	268	141	229	452	180
**Diagnosis year**							
2007-2011	4980 (16.2)	395 (15.8)	295 (5.6)	1137 (15.9)	739 (21.5)	1514 (22.9)	900 (15.6)
2012-2015	9204 (29.9)	703 (28.0)	1843 (34.7)	2218 (31.1)	900 (26.2)	1817 (27.5)	1723 (29.9)
2016-2019	9034 (29.4)	778 (31.0)	1842 (34.7)	2138 (29.9)	960 (28.0)	1698 (25.7)	1618 (28.1)
2020-2023	7538 (24.5)	631 (25.2)	1325 (25.0)	1649 (23.1)	834 (24.3)	1572 (23.8)	1527 (26.5)
**Routes to diagnosis**							
Clinically detected	12436 (40.5)	903 (36.1)	1978 (37.3)	3353 (47.1)	1287 (37.5)	2704 (41.0)	2211 (38.5)
Screening-detected	18252 (59.5)	1600 (63.9)	3323 (62.7)	3771 (52.9)	2143 (62.5)	3889 (59.0)	3526 (61.5)
Missing	68	4	4	18	3	8	31

**Tumor size ***							
T01	20767 (67.7)	1775 (70.9)	3690 (69.8)	4741 (66.5)	2197 (64.1)	4232 (64.3)	4132 (71.8)
T2	8235 (26.8)	610 (24.4)	1310 (24.8)	1962 (27.5)	1040 (30.3)	1914 (29.1)	1399 (24.3)
T3	1573 (5.1)	98 (3.9)	271 (5.1)	395 (5.5)	182 (5.3)	429 (6.5)	198 (3.4)
T4	103 (0.3)	19 (0.8)	16 (0.3)	27 (0.4)	9 (0.3)	10 (0.2)	22 (0.4)
Missing	78	5	18	17	5	16	17
**Lymph Node Status ***							
N0 (negative)	20709 (68.3)	1677 (68.4)	3595 (68.7)	4960 (69.9)	2313 (68.6)	4458 (68.8)	3706 (65.4)
N+ (positive)	9594 (31.7)	776 (31.6)	1641 (31.3)	2133 (30.1)	1058 (31.4)	2023 (31.2)	1963 (34.6)
Missing	453	54	69	49	62	120	99
**TNM stage**							
I	16985 (56.2)	1426 (58.3)	3064 (58.7)	3948 (55.8)	1807 (53.7)	3469 (53.6)	3271 (57.9)
IIA	7042 (23.3)	536 (21.9)	1109 (21.2)	1724 (24.4)	834 (24.8)	1559 (24.1)	1280 (22.6)
IIB	3744 (12.4)	281 (11.5)	592 (11.3)	907 (12.8)	458 (13.6)	818 (12.7)	688 (12.2)
IIIA	1884 (6.2)	148 (6.0)	355 (6.8)	398 (5.6)	194 (5.8)	483 (7.5)	306 (5.4)
IIIB/IIIC	574 (1.9)	57 (2.3)	100 (1.9)	100 (1.4)	73 (2.2)	137 (2.1)	107 (1.9)
Missing	527	59	85	65	67	135	116
**Histologic Grade (surgery)**							
Grade 1	7398 (24.9)	583 (23.9)	1390 (26.9)	1497 (22.1)	846 (25.3)	1666 (25.8)	1416 (25.6)
Grade 2	16879 (56.9)	1349 (55.2)	2838 (54.9)	3818 (56.5)	1934 (57.9)	3812 (59.1)	3128 (56.6)
Grade 3	5405 (18.2)	510 (20.9)	939 (18.2)	1445 (21.4)	563 (16.8)	970 (15.0)	978 (17.7)
Missing	1074	65	138	382	90	153	246
**Histologic type of BC**							
NST (formerly ductal)	23252 (75.8)	1940 (77.6)	4133 (78.0)	5572 (78.2)	2473 (72.2)	4642 (70.6)	4492 (77.9)
Lobular	4457 (14.5)	377 (15.1)	757 (14.3)	957 (13.4)	510 (14.9)	1037 (15.8)	819 (14.2)

Mixed/other	2981 (9.7)	183 (7.3)	412 (7.8)	593 (8.3)	441 (12.9)	900 (13.7)	452 (7.8)
Missing	66	7	3	20	9	22	5
**Chemotherapy use**							
Yes (alone or combination)	12759 (41.5)	1095 (43.7)	2071 (39.0)	3533 (49.5)	1454 (42.4)	2266 (34.3)	2340 (40.6)
No (other therapies)	17997 (58.5)	1412 (56.3)	3234 (61.0)	3609 (50.5)	1979 (57.6)	4335 (65.7)	3428 (59.4)
**Type of breast surgery**							
Breast conserving	21737 (70.7)	1868 (74.5)	3805 (71.7)	5190 (72.7)	2247 (65.5)	4501 (68.2)	4126 (71.5)
Mastectomy	9019 (29.3)	639 (25.5)	1500 (28.3)	1952 (27.3)	1186 (34.5)	2100 (31.8)	1642 (28.5)
**Type of axillary surgery**							
None	82 (0.3)	6 (0.2)	17 (0.3)	14 (0.2)	5 (0.1)	9 (0.1)	31 (0.5)
SN biopsy	21453 (70.2)	1714 (69.2)	3847 (73.1)	5054 (71.1)	2390 (70.0)	4534 (69.2)	3914 (68.2)
Sampling	1259 (4.1)	85 (3.4)	188 (3.6)	249 (3.5)	164 (4.8)	314 (4.8)	259 (4.5)
Axillary clearance	7757 (25.4)	672 (27.1)	1210 (23.0)	1791 (25.2)	853 (25.0)	1694 (25.9)	1537 (26.8)
Missing	205	30	43	34	21	50	27

* Tumor size and lymph node status were categorized by cT/cN at diagnosis or pT/pN after surgery, depending on treatment setting.
** Chi-square test for statistical significance of categorical variable distributions across regions were performed with all P-values < 0.001.
Abbreviation: IQR, interquartile range; NST, invasive carcinoma of no special type

Supplementary Table 4. Distribution of baseline characteristics of the study participants in those aged ≥ 65 years and according to 6 healthcare regions, as well as corresponding medical and surgical therapies

**Characteristic**	**Those aged ≥ 65 years, n (%)**	**Healthcare region, n (%)**					
		**North**	**Mid Sweden**	**Stockholm-Gotland**	**Southeast**	**West**	**South**
**Number of women**	31 179	2749 (8.8)	6042 (19.4)	6379 (20.5)	3695 (11.9)	6441 (20.7)	5873 (18.8)
**Age at diagnosis, yr**							
Median (IQR), yr	72 (68-77)	72 (68-76)	72 (68-77)	72 (68-77)	72 (69-78)	72 (68-78)	72 (68-77)
65-79	25503 (81.8)	2299 (83.6)	4959 (82.1)	5374 (84.2)	2903 (78.6)	5212 (80.9)	4756 (81.0)
≥ 80	5676 (18.2)	450 (16.4)	1083 (17.9)	1005 (15.8)	792 (21.4)	1229 (19.1)	1117 (19.0)
**Diagnosis year**							
2007-2011	4068 (13.0)	361 (13.1)	255 (4.2)	798 (12.5)	623 (16.9)	1321 (20.5)	710 (12.1)
2012-2015	9480 (30.4)	723 (26.3)	2153 (35.6)	2063 (32.3)	1060 (28.7)	1709 (26.5)	1772 (30.2)
2016-2019	9791 (31.4)	909 (33.1)	2158 (35.7)	2051 (32.2)	1071 (29.0)	1796 (27.9)	1806 (30.8)
2020-2023	7840 (25.1)	756 (27.5)	1476 (24.4)	1467 (23.0)	941 (25.5)	1615 (25.1)	1585 (27.0)
**Routes to diagnosis**							
Clinically detected	15251 (49.0)	1237 (45.1)	2805 (46.5)	3450 (54.2)	1786 (48.3)	3145 (48.9)	2828 (48.4)
Screening-detected	15856 (51.0)	1508 (54.9)	3224 (53.5)	2913 (45.8)	1908 (51.7)	3289 (51.1)	3014 (51.6)
Missing	72	4	13	16	1	7	31
**Tumor size ***							
T01	20522 (66.0)	1836 (66.9)	4044 (67.2)	4253 (66.8)	2279 (61.8)	4087 (63.6)	4023 (68.6)
T2	9097 (29.2)	778 (28.4)	1672 (27.8)	1835 (28.8)	1237 (33.5)	1985 (30.9)	1590 (27.1)
T3	1300 (4.2)	102 (3.7)	257 (4.3)	248 (3.9)	154 (4.2)	339 (5.3)	200 (3.4)
T4	190 (0.6)	28 (1.0)	45 (0.7)	31 (0.5)	18 (0.5)	18 (0.3)	50 (0.9)
Missing	70	5	24	12	7	12	10

**Lymph Node Status ***							
N0 (negative)	22191 (73.7)	1970 (74.6)	4260 (73.1)	4756 (76.1)	2624 (73.4)	4472 (73.3)	4109 (71.7)
N+ (positive)	7932 (26.3)	672 (25.4)	1567 (26.9)	1496 (23.9)	952 (26.6)	1626 (26.7)	1619 (28.3)
Missing	1056	107	215	127	119	343	145
**TNM stage**							
I	17311 (57.6)	1557 (59.0)	3386 (58.3)	3681 (59.0)	1950 (54.6)	3396 (55.8)	3341 (58.4)
IIA	7253 (24.1)	609 (23.1)	1375 (23.7)	1521 (24.4)	907 (25.4)	1489 (24.5)	1352 (23.6)
IIB	3160 (10.5)	258 (9.8)	571 (9.8)	644 (10.3)	415 (11.6)	687 (11.3)	585 (10.2)
IIIA	1595 (5.3)	150 (5.7)	305 (5.3)	278 (4.5)	209 (5.9)	368 (6.0)	285 (5.0)
IIIB/IIIC	741 (2.5)	63 (2.4)	170 (2.9)	117 (1.9)	88 (2.5)	147 (2.4)	156 (2.7)
Missing	1119	112	235	138	126	354	154
**Histologic Grade (surgery)**							
Grade 1	7340 (24.0)	684 (25.5)	1571 (26.5)	1322 (21.2)	873 (23.9)	1576 (24.7)	1314 (22.8)
Grade 2	18403 (60.1)	1550 (57.8)	3457 (58.2)	3746 (60.1)	2172 (59.5)	3986 (62.6)	3492 (60.7)
Grade 3	4882 (15.9)	449 (16.7)	907 (15.3)	1162 (18.7)	606 (16.6)	807 (12.7)	951 (16.5)
Missing	554	66	107	149	44	72	116
**Histologic type of BC**							
NST (formerly ductal)	21761 (69.9)	1982 (72.1)	4355 (72.2)	4528 (71.1)	2407 (65.3)	4342 (67.7)	4147 (70.7)
Lobular	5686 (18.3)	512 (18.6)	1090 (18.1)	1154 (18.1)	677 (18.4)	1151 (17.9)	1102 (18.8)
Mixed/other	3674 (11.8)	254 (9.2)	590 (9.8)	689 (10.8)	604 (16.4)	920 (14.3)	617 (10.5)
Missing	58	1	7	8	7	28	7
**Chemotherapy use**							
Yes (alone or combination)	4600 (14.8)	429 (15.6)	818 (13.5)	1156 (18.1)	654 (17.7)	736 (11.4)	807 (13.7)
No (other therapies)	26579 (85.2)	2320 (84.4)	5224 (86.5)	5223 (81.9)	3041 (82.3)	5705 (88.6)	5066 (86.3)

**Type of breast surgery**							
Breast conserving	20554 (65.9)	1809 (65.8)	4071 (67.4)	4579 (71.8)	2320 (62.8)	3922 (60.9)	3853 (65.6)
Mastectomy	10625 (34.1)	940 (34.2)	1971 (32.6)	1800 (28.2)	1375 (37.2)	2519 (39.1)	2020 (34.4)
**Type of axillary surgery**							
None	138 (0.5)	7 (0.3)	32 (0.5)	15 (0.2)	14 (0.4)	32 (0.5)	38 (0.7)
SN biopsy	22751 (74.9)	2023 (75.7)	4532 (77.3)	4804 (76.8)	2679 (73.8)	4470 (72.5)	4243 (73.2)
Sampling	1214 (4.0)	95 (3.6)	194 (3.3)	231 (3.7)	154 (4.2)	318 (5.2)	222 (3.8)
Axillary clearance	6281 (20.7)	547 (20.5)	1103 (18.8)	1209 (19.3)	783 (21.6)	1349 (21.9)	1290 (22.3)
Missing	795	77	181	120	65	272	80

* Tumor size and lymph node status were categorized by cT/cN at diagnosis or pT/pN after surgery, depending on treatment setting.

** Chi-square test for statistical significance of categorical variable distributions across regions were performed with all P-values < 0.001.

Abbreviation: IQR, interquartile range; NST, invasive carcinoma of no special type

**Supplementary Table 5.** Distribution of baseline characteristics of study participants in the overall cohort and according to chemotherapy use relative to timing of surgery, as well as corresponding medical and surgical therapies				
**Characteristics**	**Overall cohort n (%)**	**Stratified by setting, n (%)**		
		Neoadjuvant setting	Adjuvant setting	P-value
**Number of women**	61 935	3297 (5.3)	58 638 (94.7)	
**Healthcare region**				<0.001
North	5256 (8.5)	204 (6.2)	5052 (8.6)	
Mid Sweden	11347 (18.3)	441 (13.4)	10906 (18.6)	
Stockholm-Gotland	13521 (21.8)	1205 (36.5)	12316 (21.0)	
Southeast	7128 (11.5)	340 (10.3)	6788 (11.6)	
West	13042 (21.1)	426 (12.9)	12616 (21.5)	
South	11641 (18.8)	681 (20.7)	10960 (18.7)	
**Age at diagnosis, yr**				<0.001
Median (IQR)	*65 (54-72)*	*56 (46-68)*	*65 (55-72)*	
< 40	1588 (2.6)	362 (11.0)	1226 (2.1)	
40-49	8311 (13.4)	786 (23.8)	7525 (12.8)	
50-64	20857 (33.7)	1134 (34.4)	19723 (33.6)	
65-79	25503 (41.2)	759 (23.0)	24744 (42.2)	
≥ 80	5676 (9.2)	256 (7.8)	5420 (9.2)	
**Menopausal status**				<0.001
Premenopausal	11203 (18.6)	1240 (38.8)	9963 (17.4)	
Postmenopausal	46781 (77.5)	1868 (58.4)	44913 (78.6)	
Hysterectomy/uncertain	2374 (3.9)	90 (2.8)	2284 (4.0)	
Missing	1577	99	1478	
**Diagnosis year**				<0.001
2007-2011	9048 (14.6)	219 (6.6)	8829 (15.1)	
2012-2015	18684 (30.2)	685 (20.8)	17999 (30.7)	
2016-2019	18825 (30.4)	1194 (36.2)	17631 (30.1)	
2020-2023	15378 (24.8)	1199 (36.4)	14179 (24.2)	
**Routes to diagnosis**				<0.001
Clinically detected	27687 (44.8)	2400 (73.0)	25287 (43.2)	
Screening-detected	34108 (55.2)	887 (27.0)	33221 (56.8)	
Missing	140	10	130	
**Tumor size ***				<0.001
T01	41289 (66.8)	520 (15.8)	40769 (69.7)	
T2	17332 (28.1)	1793 (54.5)	15539 (26.6)	
T3	2873 (4.6)	791 (24.0)	2082 (3.6)	

T4	293 (0.5)	186 (5.7)	107 (0.2)	
Missing	148	7	141	
**Lymph Node Status ***				<0.001
N0 (negative)	42900 (71.0)	1419 (43.2)	41481 (72.6)	
N+ (positive)	17526 (29.0)	1862 (56.8)	15664 (27.4)	
Missing	1509	16	1493	
**TNM stage**				<0.001
I	34296 (56.9)	242 (7.4)	34054 (59.7)	
IIA	14295 (23.7)	1058 (32.3)	13237 (23.2)	
IIB	6904 (11.5)	1262 (38.5)	5642 (9.9)	
IIIA	3479 (5.8)	483 (14.8)	2996 (5.3)	
IIIB/IIIC	1315 (2.2)	229 (7.0)	1086 (1.9)	
Missing	1646	23	1623	
**Histologic Grade (surgery)**				<0.001
Grade 1	14738 (24.4)	300 (15.3)	14438 (24.7)	
Grade 2	35282 (58.5)	1337 (68.2)	33945 (58.2)	
Grade 3	10287 (17.1)	323 (16.5)	9964 (17.1)	
Missing	1628	1337	291	
**Histologic type of BC**				<0.001
NST (formerly ductal)	45013 (72.8)	2540 (78.0)	42473 (72.5)	
Lobular	10143 (16.4)	507 (15.6)	9636 (16.5)	
Mixed/other	6655 (10.8)	210 (6.4)	6445 (11.0)	
Missing	124	40	84	
**Chemotherapy use**				<0.001
No (other therapies)	44576 (72.0)	565 (17.1)	44011 (75.1)	
Yes (single or combination)	17359 (28.0)	2732 (82.9)	14627 (24.9)	
**Type of breast surgery**				<0.001
Breast conserving	42291 (68.3)	1230 (37.3)	41061 (70.0)	
Mastectomy	19644 (31.7)	2067 (62.7)	17577 (30.0)	
**Type of axillary surgery**				<0.001
None	220 (0.4)	12 (0.4)	208 (0.4)	
SN biopsy	44204 (72.5)	713 (22.2)	43491 (75.3)	
Sampling	2473 (4.1)	222 (6.9)	2251 (3.9)	
Axillary clearance	14038 (23.0)	2268 (70.5)	11770 (20.4)	
Missing	1000	82	918	

* Tumor size and lymph node status were categorized by cT/cN at diagnosis or pT/pN after surgery, depending on treatment setting.
Abbreviation: IQR, interquartile range; NST, invasive carcinoma of no special type

Supplementary Table 6. Overall chemotherapy administration according to healthcare regions in the entire cohort and settings, respectively

Healthcare region	Number of patients, N	Chemotherapy user, n (%)		
		Overall [a]	Neoadjuvant setting [b]	Adjuvant setting [b]
North	5256	1524 (29.0)	151 (9.9)	1373 (90.1)
Mid Sweden	11347	2889 (25.5)	331 (11.5)	2558 (88.5)
Stockholm-Gotland	13521	4689 (34.7)	1059 (22.6)	3630 (77.4)
Southeast	7128	2108 (29.6)	283 (13.4)	1825 (86.6)
West	13042	3002 (23.0)	351 (11.7)	2651 (88.3)
South	11641	3147 (27.0)	557 (17.7)	2590 (82.3)

[a] % chemotherapy user = n-overall user / N-patients
[b] % chemotherapy user = n-setting user / n-overall user

**Supplementary Table 7.** Univariate and multivariable analyses of factors associated with chemotherapy use in the overall cohort, measured using both crude and adjusted odds ratios (ORs)		
**Factor**	**Receipt of chemotherapy in overall cohort**	
	**Crude OR (95% CI)**	**Adjusted OR (95% CI)**
**Age group, yr**		
< 40	1 [Reference]	1 [Reference]
40-49	0.29 (0.25-0.33)	0.33 (0.28-0.39)
50-64	0.14 (0.12-0.16)	0.16 (0.14-0.19)
65-79	0.06 (0.05-0.07)	0.053 (0.046-0.062)
≥ 80	0.004 (0.003-0.005)	0.001 (0.0006-0.0012)
**Diagnosis year**		
2007-2011	1 [Reference]	1 [Reference]
2012-2015	1.17 (1.11-1.24)	1.63 (1.51-1.76)
2016-2019	1.22 (1.15-1.29)	1.72 (1.59-1.87)
2020-2023	1.24 (1.17-1.32)	1.77 (1.63-1.92)
**Routes to diagnosis**		
Clinically detected	1 [Reference]	1 [Reference]
Screening-detected	0.60 (0.58-0.63)	0.84 (0.80-0.88)
**Tumour size ***		
T01	1 [Reference]	1 [Reference]
T2	3.87 (3.72-4.02)	3.82 (3.61-4.03)
T3	8.20 (7.57-8.89)	6.14 (5.42-6.96)
T4	5.10 (4.05-6.43)	2.44 (1.65-3.61)
**Lymph Node Status ***		
Negative	1 [Reference]	1 [Reference]
Positive	7.72 (7.42-8.03)	8.40 (7.98-8.85)
**Histologic Grade (surgery)** [1]		
Grade 1	1 [Reference]	NA
Grade 2	4.31 (4.02-4.63)	NA
Grade 3	21.6 (20.0-23.4)	NA
**Histologic type of BC**		
NST (formerly ductal)	1 [Reference]	1 [Reference]
Lobular	0.88 (0.84-0.93)	0.61 (0.57-0.66)

Mixed/other	0.53 (0.49-0.56)	0.55 (0.50-0.60)
**Healthcare region [2]**		
Mid Sweden	1 [Reference]	1 [Reference]
North	1.20 (1.11-1.29)	1.41 (1.28-1.56)
Stockholm-Gotland	1.55 (1.47-1.64)	1.64 (1.52-1.76)
Southeast	1.23 (1.15-1.31)	1.42 (1.29-1.55)
West	0.88 (0.83-0.93)	0.78 (0.72-0.84)
South	1.08 (1.02-1.15)	1.02 (0.94-1.11)
**Timing relative to surgery**		
Neoadjuvant setting	1 [Reference]	1 [Reference]
Adjuvant setting	0.07 (0.06-0.08)	0.13 (0.11-0.15)
**C-statistic for aera under ROC**	NA	**0.88**

[1] Grade was not available for patients in neoadjuvant setting.
[2] Healthcare region was treated as one proxy factor of region-level variation.
*Tumor size and lymph node status were categorized by cT/cN at diagnosis or pT/pN after surgery, depending on treatment setting.
Abbreviation: NST, invasive carcinoma of no special type

Supplementary Table 8a. Based on BcBaSe3.0 database with patients diagnosed between 2008-2019, odds ratios (ORs) and 95% CI were estimated comparing chemotherapy use between healthcare regions.

**Healthcare regions compared**	**Univariate analysis,** crude ORs (95% CIs)	**Multivariable Model 1,** adjusted ORs (95%CIs)	**Multivariable Model 2,** adjusted ORs (95%CIs)	**Multivariable Model 3,** adjusted ORs (95%CIs)
North / Mid Sweden	1.43 (1.30-1.57)	1.50 (1.32-1.72)	1.50 (1.31-1.71)	1.49 (1.30-1.70)
Stockholm-Gotland / Mid Sweden	1.18 (1.10-1.27)	1.31 (1.20-1.44)	1.26 (1.14-1.39)	1.23 (1.11-1.36)
Southeast / Mid Sweden	1.07 (0.98-1.17)	1.36 (1.20-1.54)	1.37 (1.21-1.55)	1.37 (1.21-1.55)
West / Mid Sweden	0.75 (0.70-0.82)	0.73 (0.66-0.82)	0.73 (0.66-0.82)	0.73 (0.65-0.81)
South / Mid Sweden	1.06 (0.98-1.15)	0.92 (0.82-1.02)	0.92 (0.83-1.02)	0.92 (0.82-1.02)
Stockholm-Gotland / North	0.82 (0.75-0.90)	0.86 (0.76-0.97)	0.83 (0.73-0.94)	0.81 (0.71-0.92)
Southeast / North	0.75 (0.67-0.83)	0.88 (0.77-1.01)	0.89 (0.78-1.02)	0.90 (0.78-1.03)
West / North	0.53 (0.48-0.58)	0.47 (0.41-0.53)	0.47 (0.41-0.53)	0.47 (0.41-0.53)
South / North	0.74 (0.68-0.82)	0.60 (0.53-0.68)	0.60 (0.53-0.68)	0.60 (0.53-0.69)
Southeast / Stockholm-Gotland	0.91 (0.84-0.98)	1.02 (0.91-1.14)	1.05 (0.94-1.18)	1.09 (0.97-1.22)
West / Stockholm-Gotland	0.64 (0.60-0.69)	0.53 (0.48-0.58)	0.55 (0.50-0.60)	0.56 (0.50-0.61)
South / Stockholm-Gotland	0.90 (0.84-0.97)	0.68 (0.62-0.75)	0.71 (0.64-0.78)	0.73 (0.66-0.80)
West / Southeast	0.70 (0.65-0.77)	0.50 (0.45-0.56)	0.50 (0.44-0.56)	0.49 (0.44-0.55)
South / Southeast	1.00 (0.91-1.09)	0.63 (0.56-0.71)	0.63 (0.56-0.70)	0.63 (0.56-0.70)
South / West	1.41 (1.30-1.52)	1.22 (1.10-1.35)	1.22 (1.10-1.35)	1.23 (1.11-1.36)

Model 1 was adjusted for age at diagnosis, year of diagnosis, T stage, N stage, joint factor, histologic grade and menopausal status.
Model 2 was further adjusted for level of education
Model 3 was even further adjusted for the individual sub-component of the disposable income of the household in quintiles.

Abbreviations: HRs, hazard ratios; CIs, confidence intervals.

Supplementary Table 8b. Based on BcBaSe3.0 database with patients diagnosed between 2008-2019, hazard ratios (HRs) and 95% CI between regions were estimated based on sequential models adjusting for socioeconomic factors.

**Healthcare region**	**Univariate analysis,** crude HRs (95% CIs)	**Multivariable Model 1,** adjusted HRs (95%CIs)	**Multivariable Model 2,** adjusted HRs (95%CIs)	**Multivariable Model 3,** adjusted HRs (95%CIs)
Southeast	1 (reference)	1 (reference)	1 (reference)	1 (reference)
North	0.96 (0.80-1.14)	0.91 (0.77-1.09)	0.91 (0.77-1.09)	0.92 (0.88-1.10)
Mid Sweden	1.04 (0.90-1.19)	0.97 (0.84-1.12)	0.97 (0.84-1.12)	0.98 (0.85-1.13)
Stockholm-Gotland	0.57 (0.49-0.66)	0.63 (0.55-0.73)	0.69 (0.60-0.80)	0.72 (0.63-0.84)
West	0.96 (0.84-1.09)	0.98 (0.86-1.12)	1.00 (0.88-1.15)	1.01 (0.88-1.15)
South	0.75 (0.65-0.87)	0.73 (0.63-0.85)	0.75 (0.64-0.87)	0.75 (0.65-0.87)

Model 1 was adjusted for age at diagnosis, year of diagnosis, T stage, N stage, joint factor, histologic grade and menopausal status.
Model 2 was further adjusted for level of education
Model 3 was even further adjusted for the individual sub-component of the disposable income of the household in quintiles.

Abbreviations: HRs, hazard ratios; CIs, confidence intervals.

**Supplementary Table 9.** Number and percentage of patients with missing ER/HER2-status, not operated or missing ***among those 132739 women with unilateral breast cancer***, by healthcare region and diagnosis year				
**Diagnosis year**	**Healthcare Region**	**Missing ER-status n (%)**	**Missing HER2 n (%)**	**Not operated or missing n (%)**
2007-2023 (overall)	North	1663 (14.6)	1911 (16.7)	1096 (9.6)
	Mid Sweden	4061 (14.8)	4650 (16.9)	2409 (8.8)
	Stockholm-Gotland	4582 (15.2)	5596 (18.5)	2872 (9.5)
	Southeast	2024 (14.8)	2362 (17.3)	1230 (9.0)
	West	3599 (14.8)	4000 (16.5)	2456 (10.1)
	South	4021 (15.6)	5758 (22.4)	2589 (10.1)
2011-2022 (subgroup 2)	North	1163 (13.6)	1298 (15.1)	768 (9.0)
	Mid Sweden	2938 (14.1)	3228 (15.4)	1665 (8.0)
	Stockholm-Gotland	3121 (14.1)	3653 (16.5)	1856 (8.4)
	Southeast	1434 (13.7)	1691 (16.1)	858 (8.2)
	West	2389 (13.3)	2681 (14.9)	1611 (9.0)
	South	2801 (14.7)	3357 (17.7)	1831 (9.6)

Supplementary Table 10. Results of the sensitivity analysis comparing proportion of chemotherapy use, and 10-year cumulative risk of all-cause mortality among patients diagnosed in 2011-2022, or 2007-2023, stratified by healthcare regions

	**The entire cohort (2007-2023)**	**Sub-cohort of patients with more comprehensive treatment data (2011-2022)**
**Chemotherapy use, n (%)**		
**Overall**	17359 (28.0%)	15689 (28.8%)
North	1524 (29.0)	1338 (29.4)
Mid Sweden	2889 (25.5)	2776 (25.4)
Stockholm-Gotland	4689 (34.7)	4297 (35.1)
Southeast	2108 (29.6)	1813 (30.9)
West	3002 (23.0)	2585 (24.6)
South	3147 (27.0)	2880 (27.9)
**Endocrine therapy (alone or in combination with others), n (%)**		
**Overall**	55890 (90.2)	49170 (90.4)
Endo + Chemo + RT	15033 (26.9)	13641 (27.7)
Endo + Chemo	1895 (3.4)	1683 (3.4)
Endo + RT	27347 (48.9)	24058 (48.9)
Endo only	11615 (20.8)	9788 (19.9)
**Across regions**		
North	9840 (86.7)	9460 (86.7)
Mid Sweden	4451 (84.7)	3887 (85.4)
Stockholm-Gotland	13223 (97.8)	11966 (97.9)
Southeast	6700 (94.0)	5545 (94.5)
West	11945 (91.6)	9696 (92.2)
South	9731 (83.6)	8616 (83.6)
**Unadjusted 10-year cumulative risk of mortality (95% CIs)**		
North	22.7% (21.1-24.3)	22.9% (21.0-24.7)
Mid Sweden	22.0% (20.9-23.1)	22.0% (20.9-23.2)
Stockholm-Gotland	18.2% (17.3-19.1)	18.2% (17.3-19.2)
Southeast	22.2% (20.9-23.5)	21.6% (20.1-23.1)
West	23.2% (22.2-24.1)	22.0% (20.8-23.1)
South	20.1% (19.1-21.1)	20.1% (19.0-21.2)

Supplementary Table 11. Regional prognosis difference presented with hazard ratios and 95% confidence intervals, with Southeast healthcare region as the reference, from univariate analysis, multivariable model 1 (with adjustment for patient and tumor characteristics), and Model 2 (adjusting for both baseline covariates and treatments), respectively.

**Healthcare region**	**The entire cohort** (2007-2023)	**Sub-cohort of pts with comprehensive treatment data** (2011-2022)
**Univariate analysis,** crude HRs and 95% CIs		
Southeast	1 (reference)	1 (reference)
North	1.01 (0.92-1.11)	1.03 (0.92-1.14)
Mid Sweden	0.97 (0.90-1.05)	0.98 (0.90-1.07)
Stockholm-Gotland	0.77 (0.71-0.84)	0.79 (0.72-0.86)
West	1.01 (0.94-1.09)	0.98 (0.89-1.07)
South	0.88 (0.81-0.95)	0.88 (0.81-0.97)
**Multivariable Model 1,** adjusting for patient and tumor baseline characteristics		
Southeast	1 (reference)	1 (reference)
North	1.04 (0.94-1.15)	1.09 (0.97-1.22)
Mid Sweden	0.94 (0.86-1.02)	0.96 (0.88-1.06)
Stockholm-Gotland	0.80 (0.74-0.87)	0.83 (0.75-0.91)
West	1.02 (0.94-1.11)	1.04 (0.95-1.14)
South	0.89 (0.81-0.96)	0.90 (0.82-0.99)
**Multivariable Model 2**, with adjustment for baseline characteristics and treatments		
Southeast	1 (reference)	1 (reference)
North	1.07 (0.96-1.18)	1.10 (0.98-1.23)
Mid Sweden	0.96 (0.88-1.05)	0.98 (0.89-1.07)
Stockholm-Gotland	0.84 (0.77-0.91)	0.85 (0.78-0.94)
West	1.04 (0.96-1.12)	1.04 (0.95-1.15)
South	0.88 (0.81-0.96)	0.89 (0.81-0.98)

Supplementary Fig 1. Estimated absolute difference in proportions of chemotherapy use between healthcare regions among patients aged < 65 at diagnosis, based on post-hoc pairwise comparisons. The vertical line represents the null value (difference =0), indicating no difference between the compared regions.

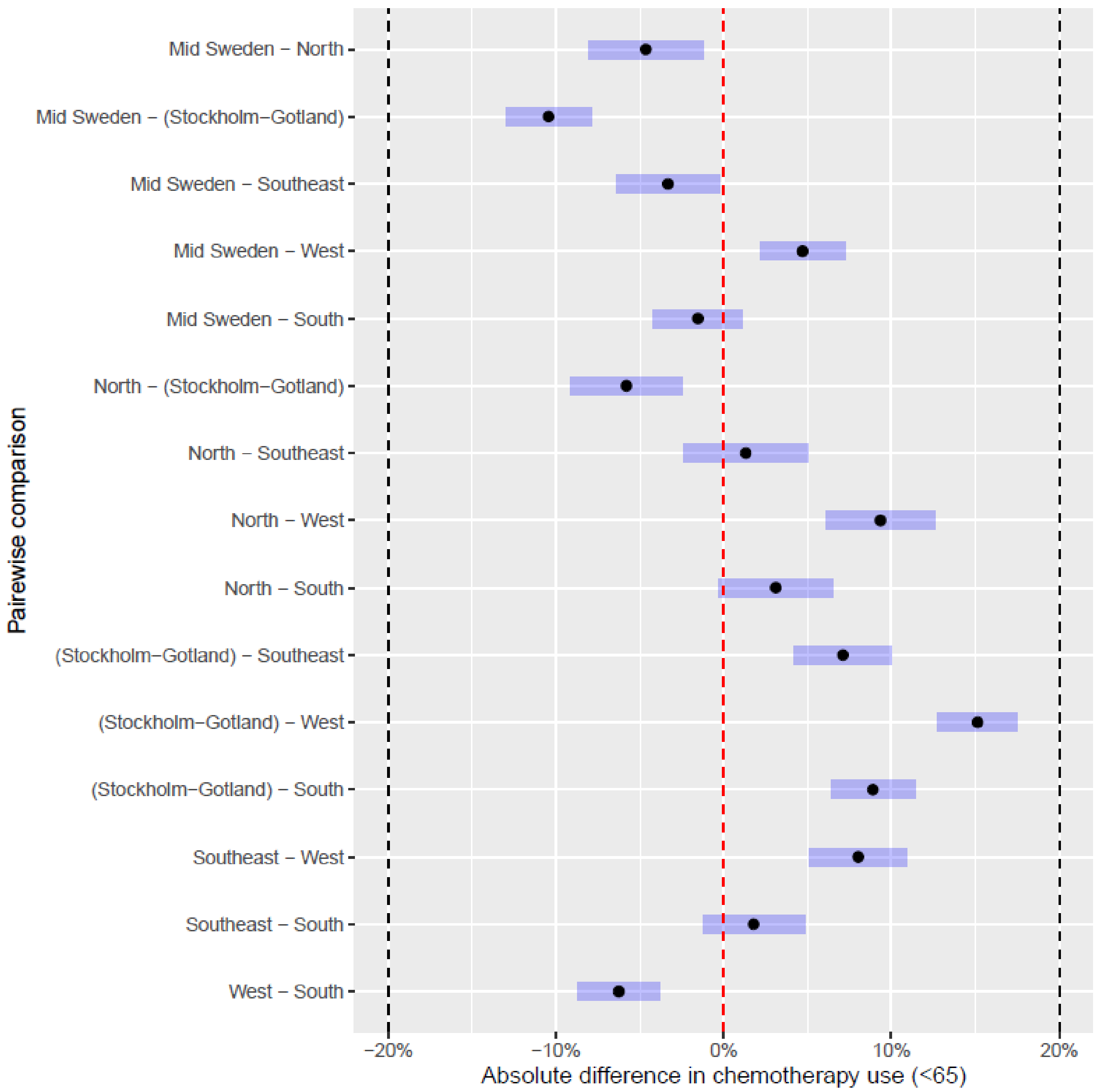

Supplementary Fig 2. Estimated absolute difference in proportions of chemotherapy use between healthcare regions among patients aged 65 years or older at diagnosis, based on post-hoc pairwise comparisons. The vertical line represents the null value (difference =0), indicating no difference between the compared regions.

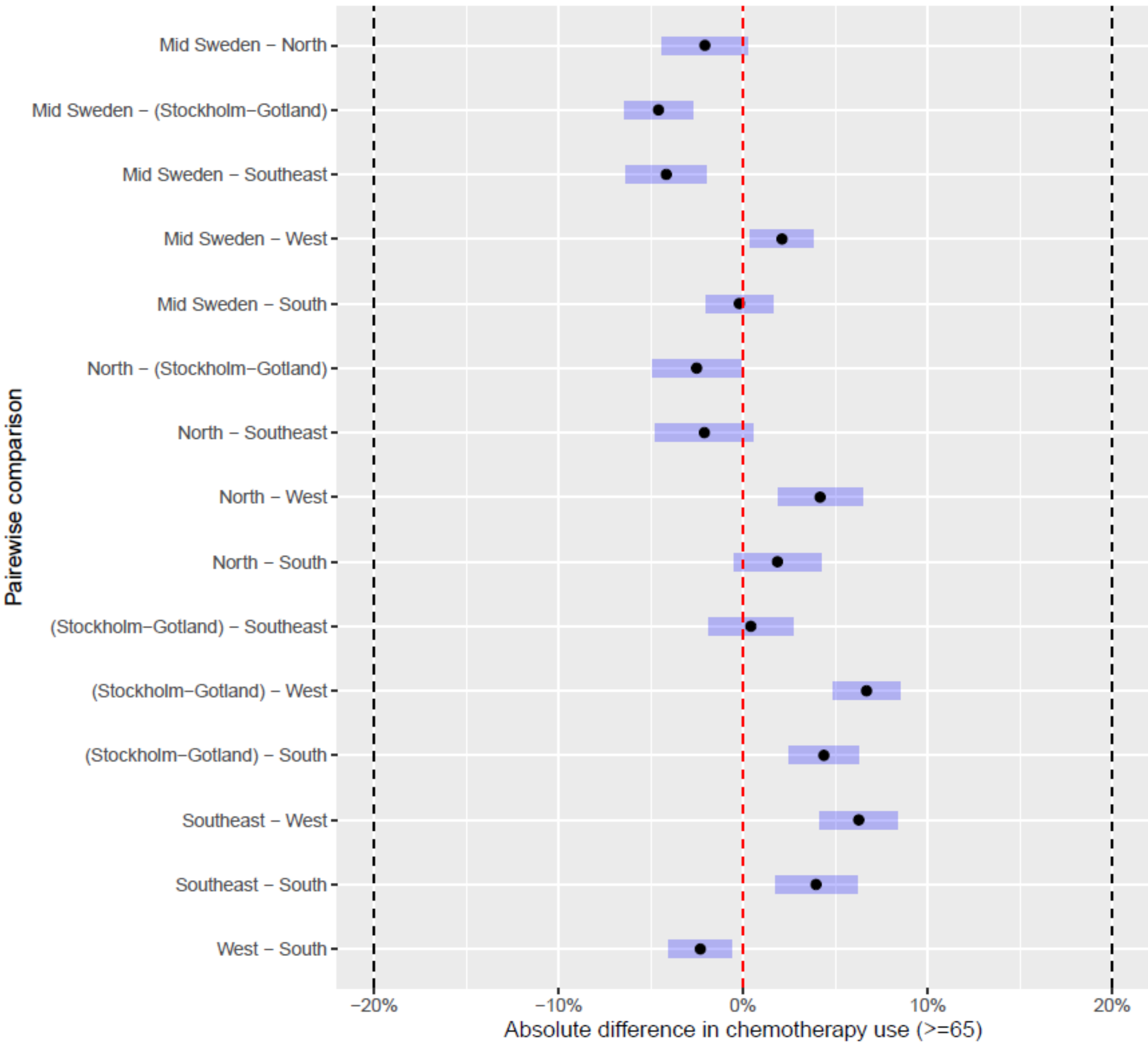

Supplementary Fig 3. Proportion of chemotherapy use according to age of diagnosis and healthcare regions.

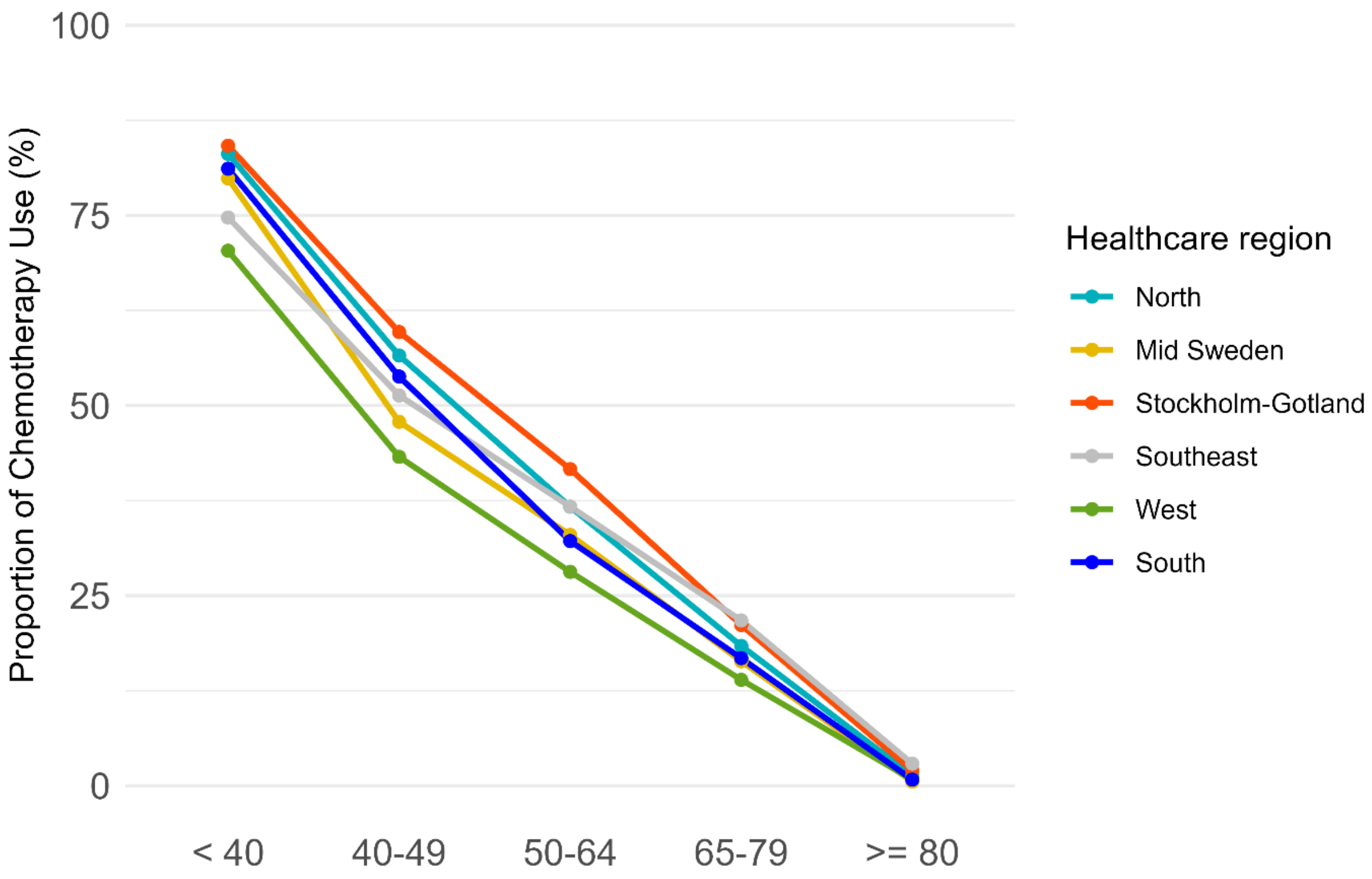

Supplementary Fig 4. Proportion of chemotherapy use according to TNM stage and healthcare regions.

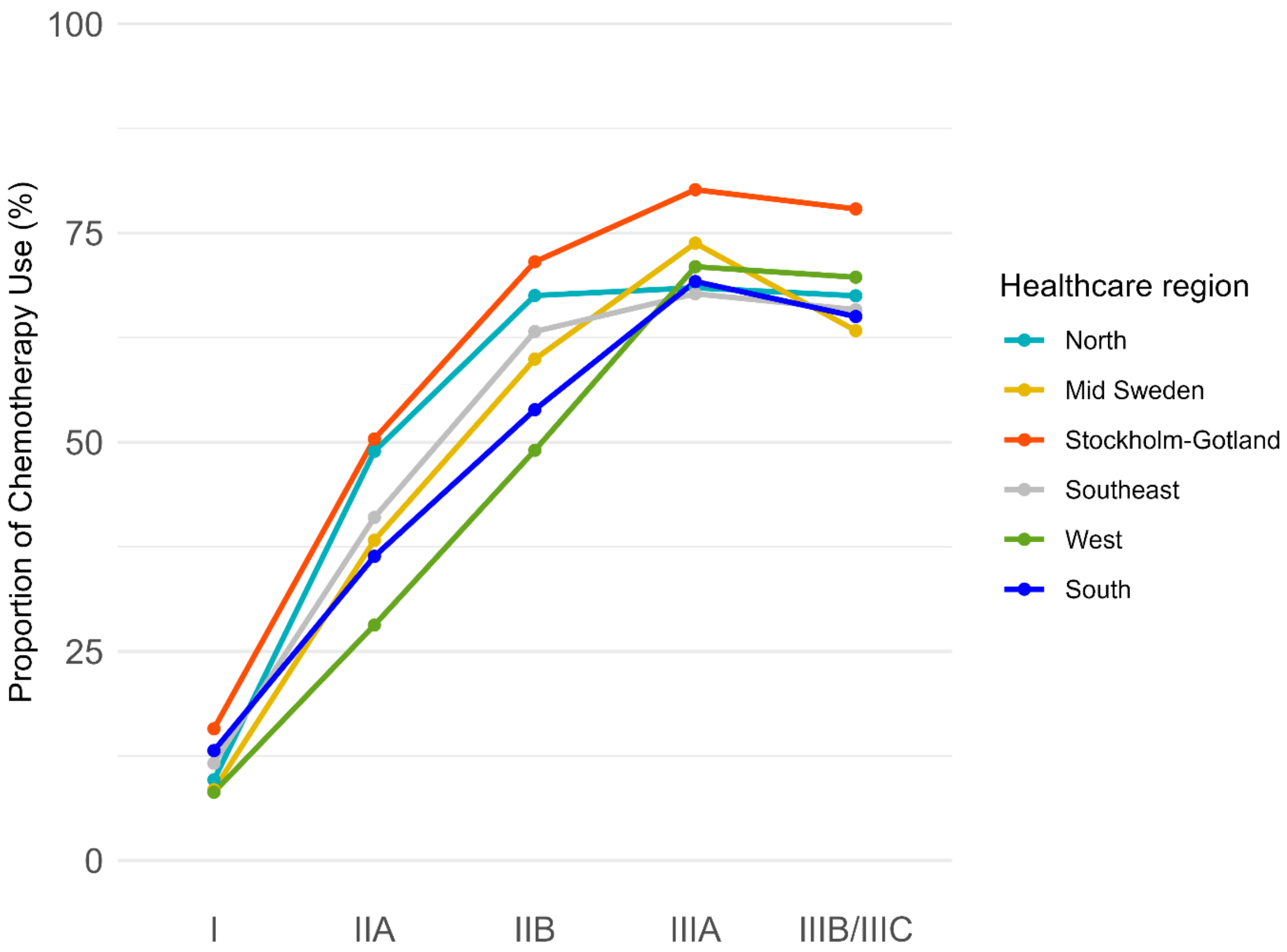

Supplementary Fig 5. Proportion of chemotherapy use according to histological grade and healthcare regions.

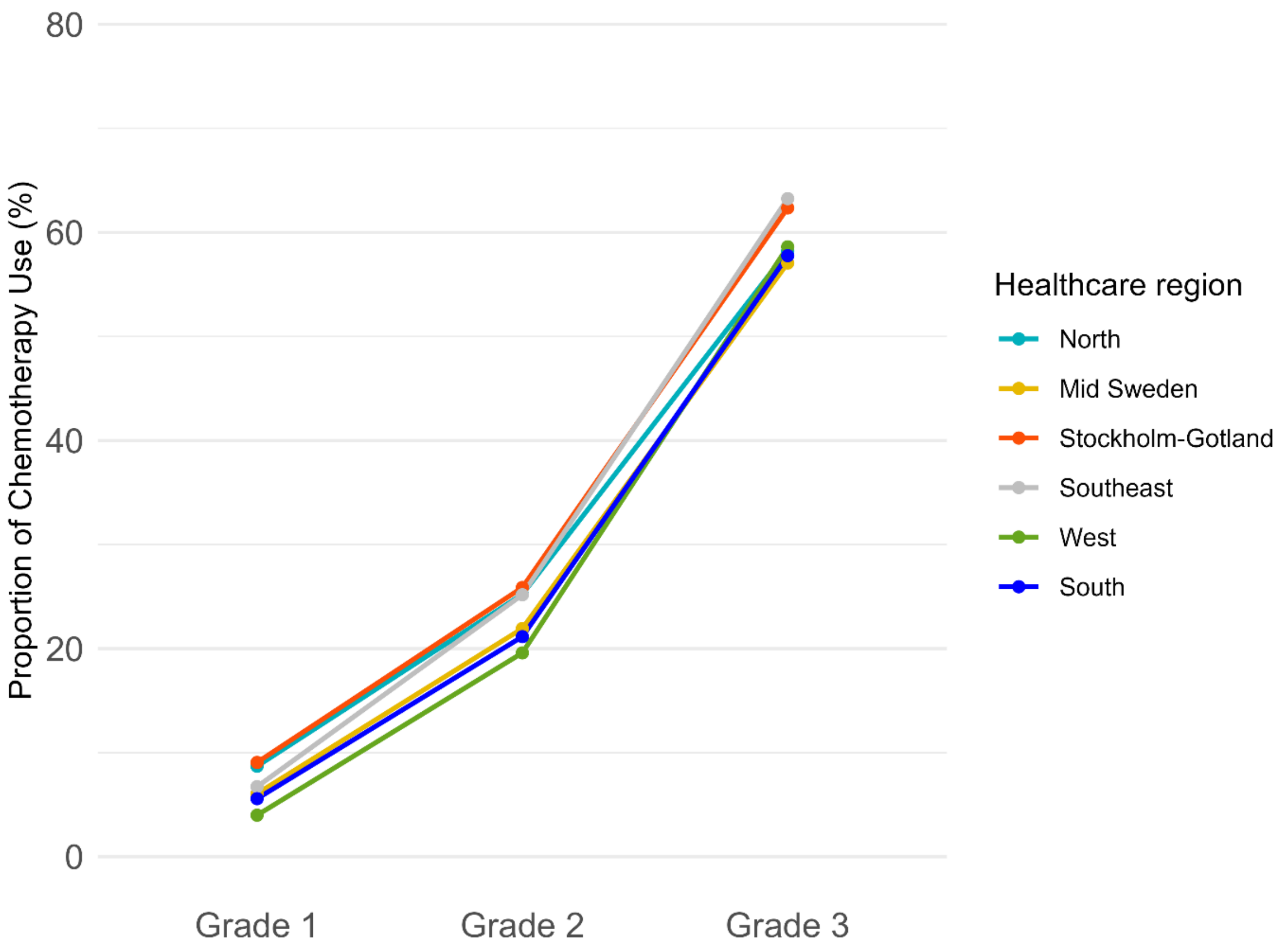

Supplementary Fig 6. Proportion of chemotherapy use according to year of diagnosis and healthcare regions.

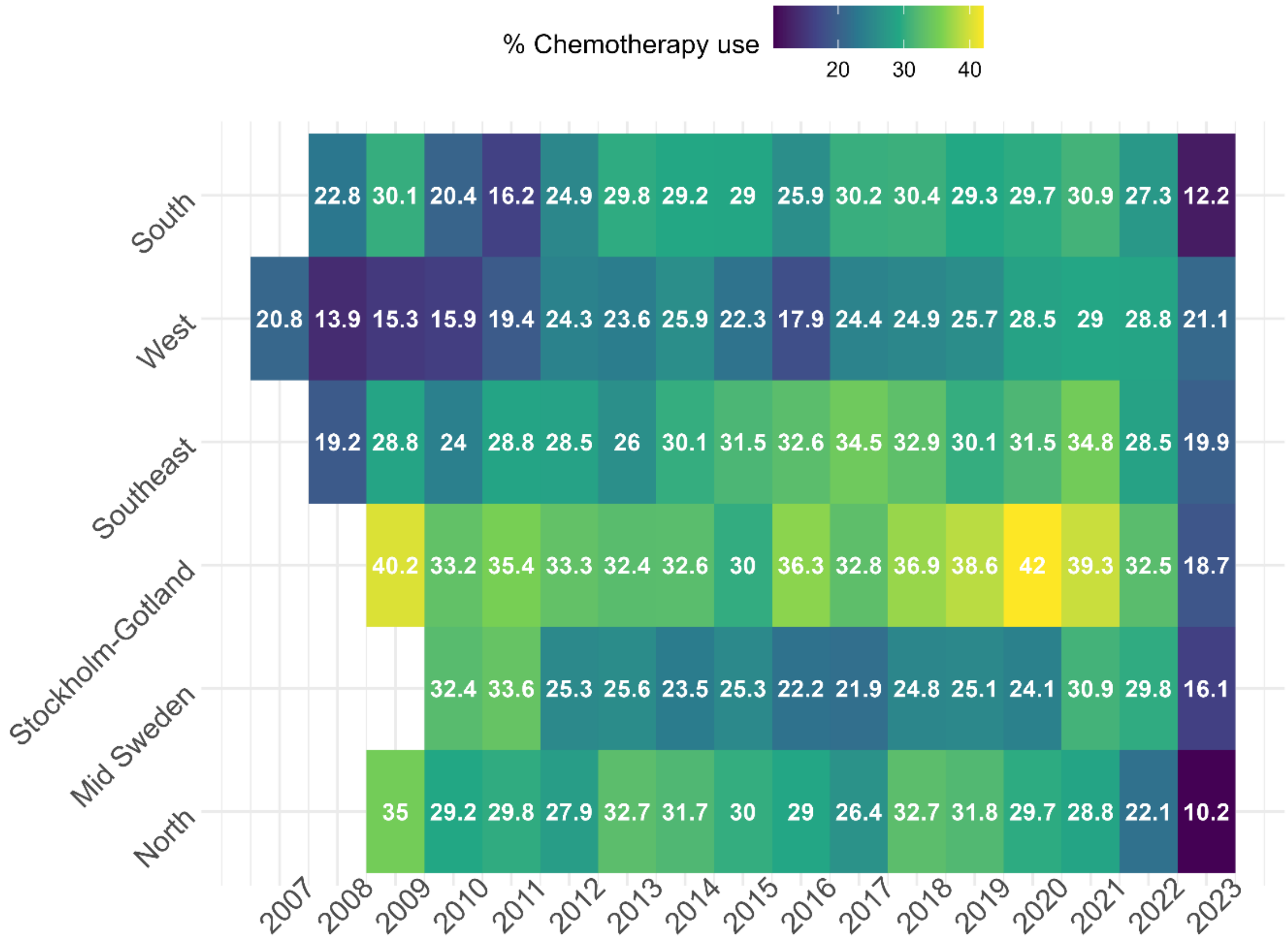

* Presented when each cell with the total number of pts > 50

Supplementary Fig 7. Adjusted (or marginal) cumulative risk and incidence rates of all-cause mortality (after adjusting for patient and tumor covariates) in the entire cohort.

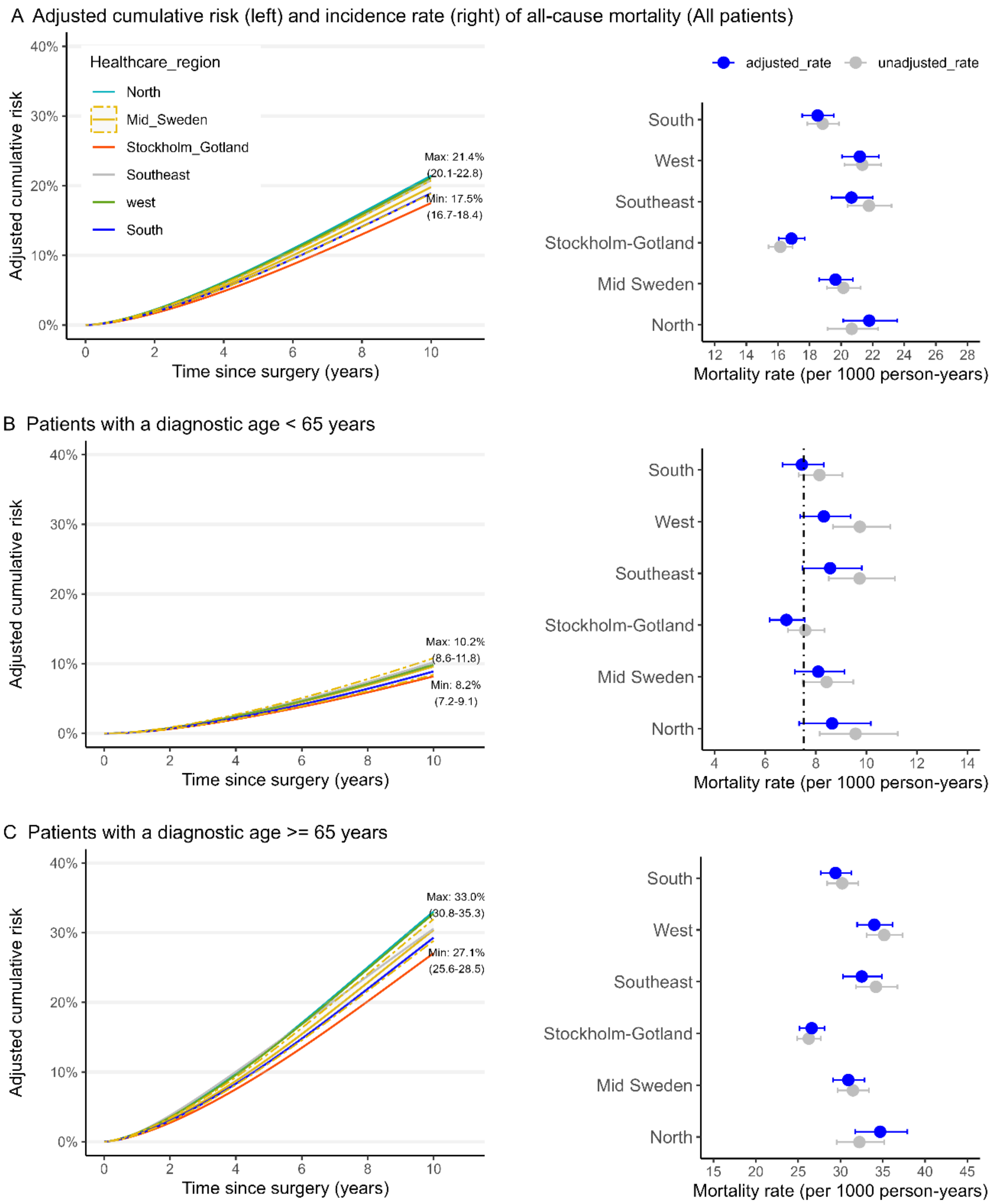

Supplementary Fig 8. Among all unilateral breast cancer, excluding synchronous contralateral BC illustrated in A, plots of B-D present the percentage of patients diagnosed with ER-positive, HER2-negative, and operated by year of diagnosis (after excluding missing).

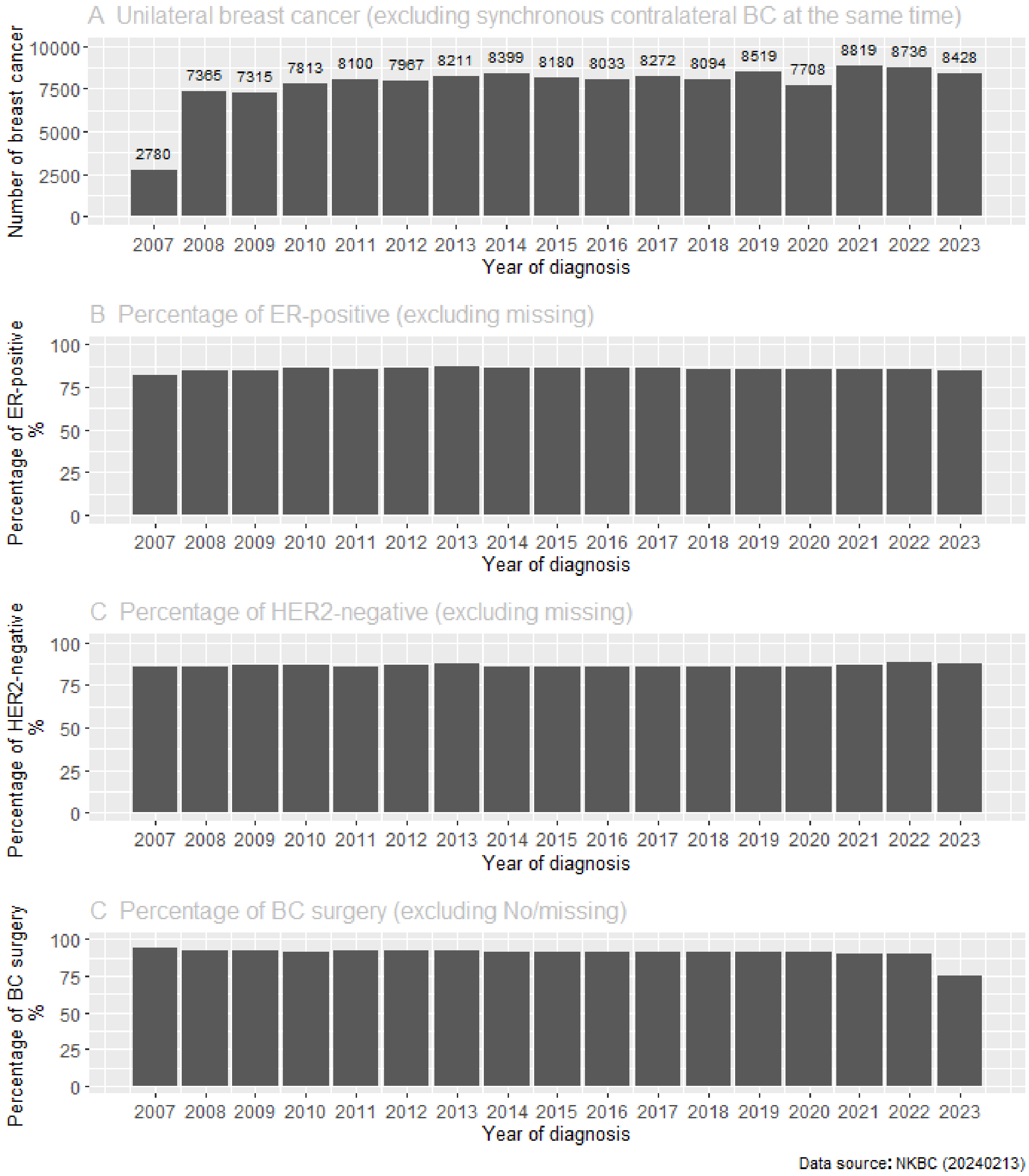

Supplementary Fig 9. Unadjusted cumulative risk of mortality before excluding patients with missing treatment data were estimated by healthcare regions (among 73911 individuals illustrated in Fig 1).

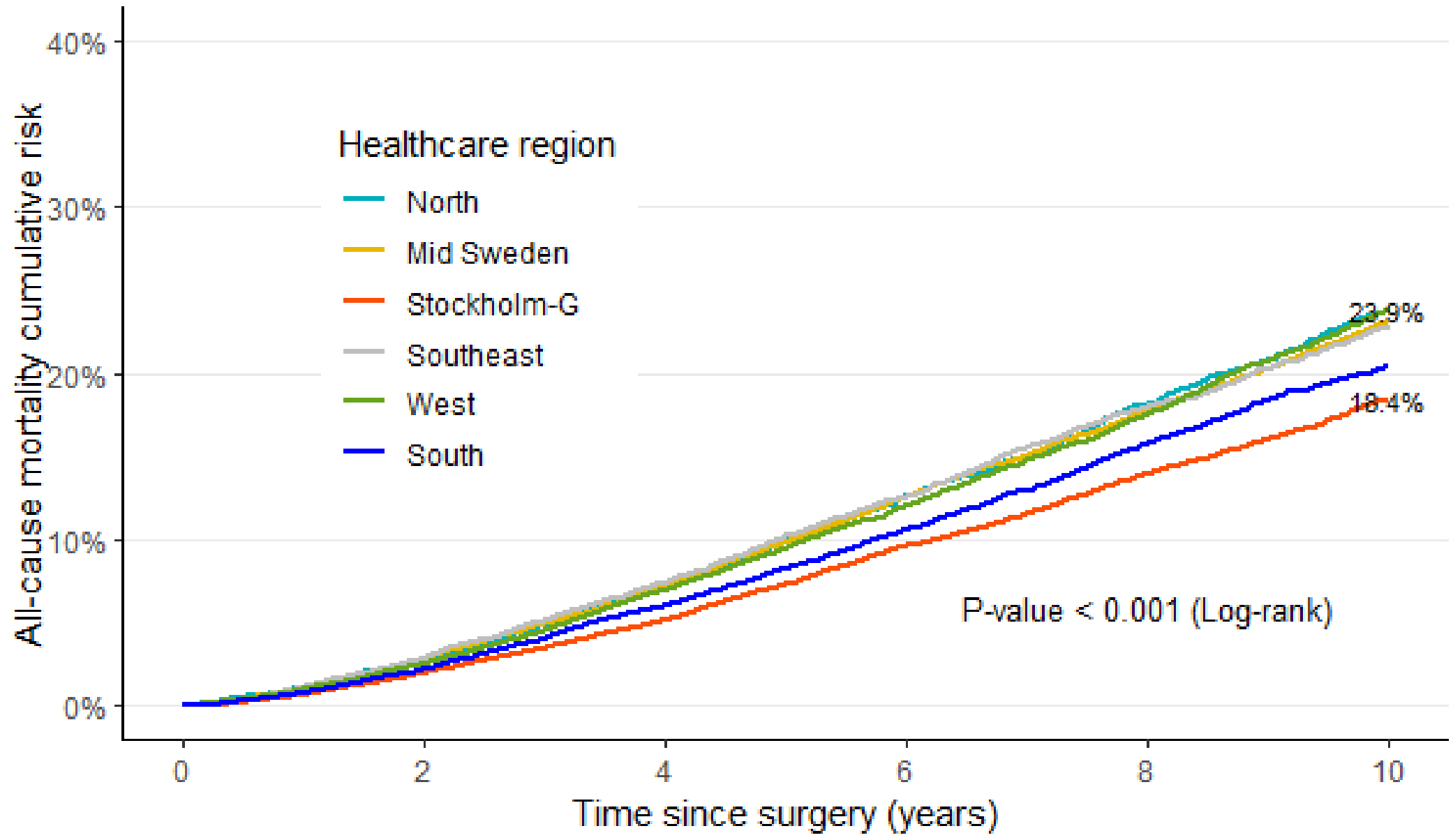

North	6110	5139	4127	3142	2200	1446
Mid Sweden	15558	13153	10599	8189	5950	3937
Stockholm-G	17038	14494	11999	9550	7198	5079
Southeast	7941	6704	5381	4066	3031	2053
West	14291	12267	9934	7809	5826	3953
South	12973	10824	8670	6688	4796	3032

Supplementary Fig 10. Adjusted (or marginal) cumulative risk and incidence rates of all-cause mortality (after adjusting for covariates and treatments) in the sub-cohort of pts diagnosed between 2011-2022.

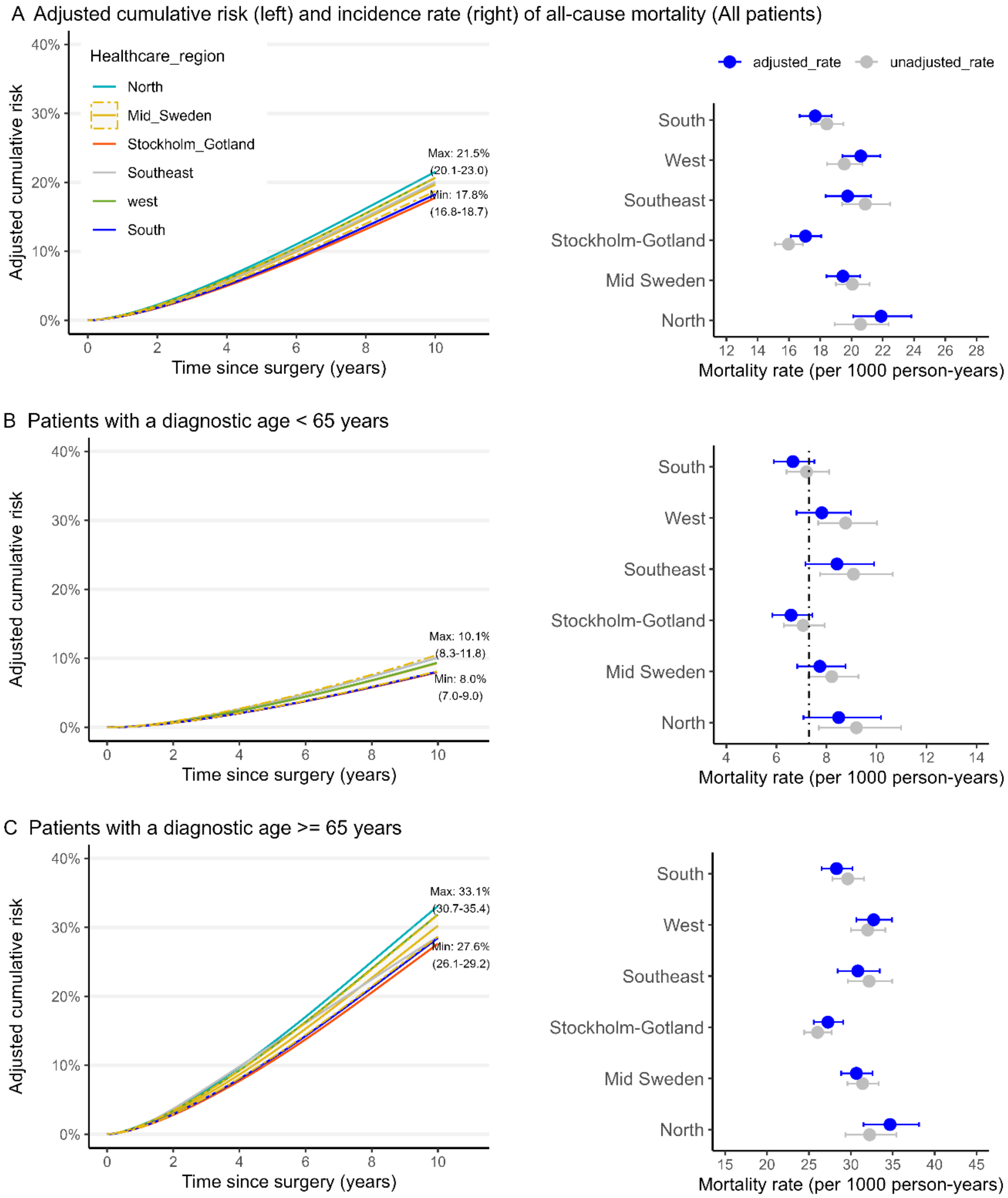

Supplementary Fig 11. Observed cumulative risk of all-cause mortality across healthcare regions, stratified by menopausal status: (A) Pre-menopausal women and (B) Post-menopausal women.

A

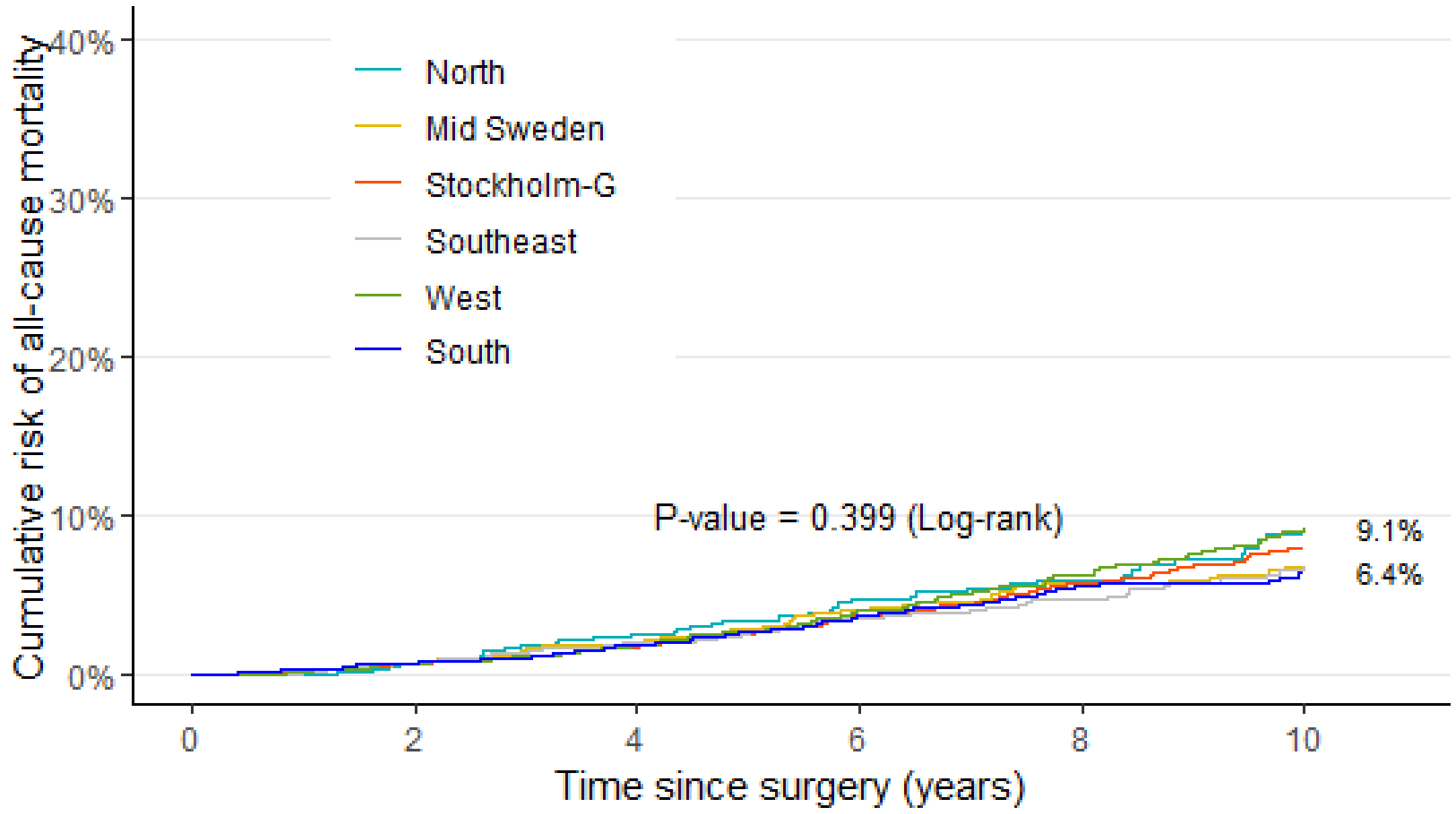

806	717	578	435	313	220
1865	1690	1357	1025	693	386
3092	2823	2307	1771	1308	837
1102	970	786	626	485	334
2210	1939	1595	1312	992	681
2128	1866	1520	1213	877	550

B

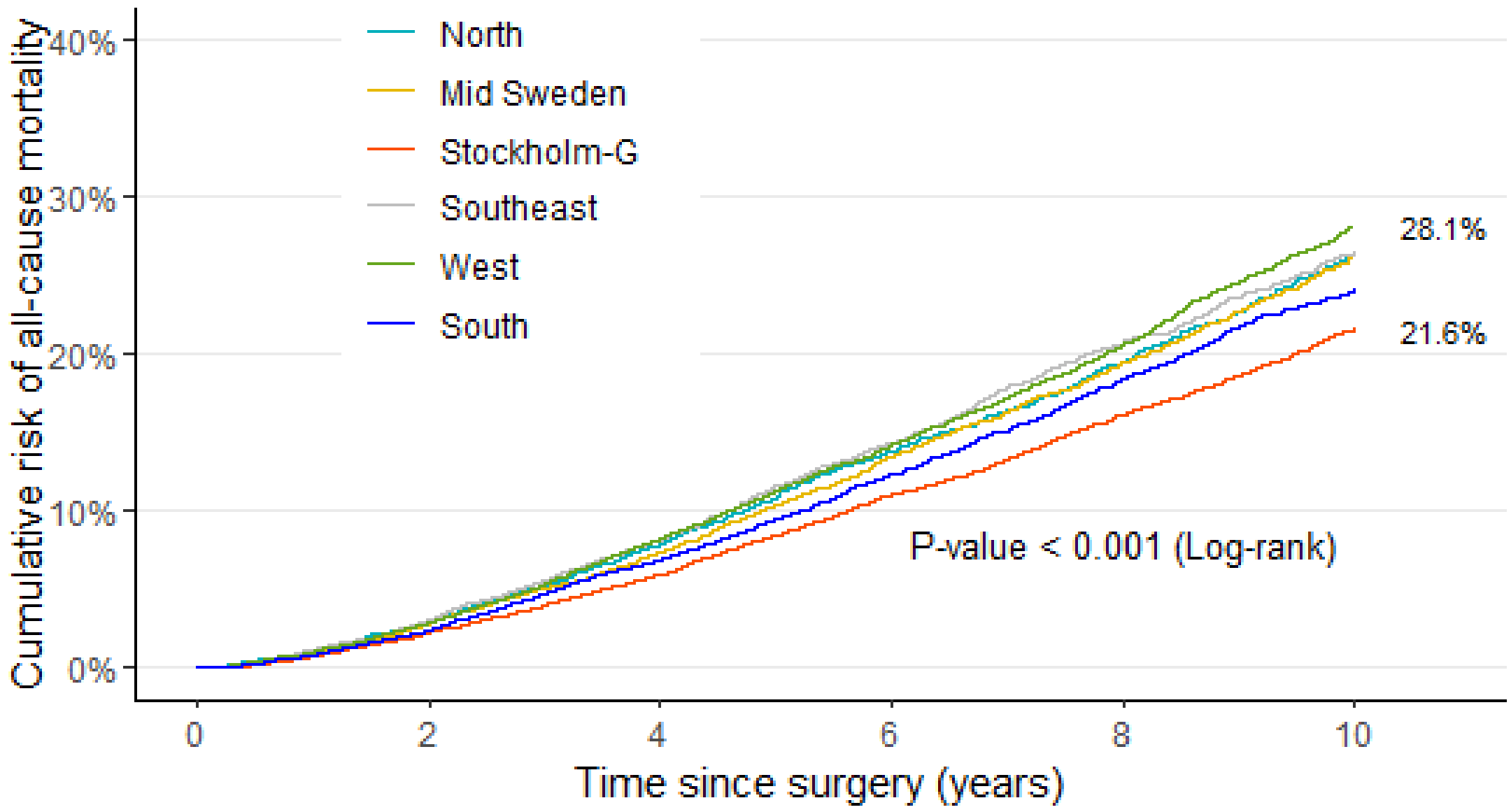

4162	3576	2827	2095	1388	820
8914	7980	6174	4403	2772	1380
10027	8982	7219	5518	3842	2372
5512	4815	3801	2784	2002	1299
9264	7966	6265	4690	3232	1960
8902	7627	6045	4536	3156	1902
